# TRIM47 Regulates Energy Metabolism via Glycolytic Reprogramming to Drive Hepatocellular Carcinoma Progression and Represents an Efficient Therapeutic Target

**DOI:** 10.1002/advs.202416996

**Published:** 2026-02-03

**Authors:** Weijie Sun, Yihang Yuan, Qian Qiu, Kexuan Tan, Xutong Li, Haotian Li, Luyang Kang, Yuting Gu, Ziheng Zhang, Jiayu He, Jiali Li, Junjie Lin, Zihan Xie, Kexing Han, Jiabin Li, Yang Zhang, Ting Wu, Yufeng Gao

**Affiliations:** ^1^ Department of Infectious Disease The First Affiliated Hospital of Anhui Medical University Hefei China; ^2^ Department of Medical Oncology First Affiliated Hospital of Bengbu Medical University Bengbu China; ^3^ Anhui Province Key Laboratory of Infectious Diseases Anhui Medical University Hefei China; ^4^ Department of General Surgery Nanjing Drum Tower Hospital Affiliated Hospital of Medical School Nanjing University Nanjing China; ^5^ Department of Pathology The First Affiliated Hospital of Anhui Medical University Hefei China; ^6^ Department of Immunology School of Basic Medical Sciences Anhui Medical University Hefei China; ^7^ School of Life Sciences Jiangsu University Zhenjiang China; ^8^ Center for Nanomedicine and Department of Anesthesiology Perioperative and Pain Medicine Brigham and Women's Hospital Harvard Medical School Boston USA

**Keywords:** energy metabolism regulation, glycolytic reprogramming, hepatocellular carcinoma, siRNA therapy, TRIM47

## Abstract

Hepatocellular carcinoma (HCC) is an aggressive cancer with limited therapeutic targets and a poor prognosis. Aberrant energy metabolism plays a pivotal role in HCC progression by fulfilling the energy demands of rapidly proliferating tumor cells. In this study, tripartite motif‐containing protein 47 (TRIM47) was found to be significantly upregulated in patient‐derived HCC tissues, with clinical data revealing that higher TRIM47 expression correlates with poorer patient outcomes. Mechanistic investigations demonstrated that TRIM47 remodels energy metabolism by glycolytic reprogramming through its interaction with the K51 site of fructose‐1,6‐bisphosphatase (FBP1) through K48‐linked ubiquitination, thereby promoting HCC proliferation and tumor metastasis. Rescue experiments and bortezomib intervention experiments further confirmed that FBP1 is essential for mediating the oncogenic effects of TRIM47 in HCC progression. To explore its therapeutic potential, TRIM47 siRNA was developed and loaded into poly (lactic acid)‐DC‐Chol nanoparticles (siTRIM47@PD NPs), which significantly reduced tumor growth and metastasis in an orthotopic HCC animal model, highlighting the potential of TRIM47 as a therapeutic target. Together, these findings underscore the pivotal role of TRIM47 in HCC progression through FBP1‐mediated regulation of energy metabolism, and highlight siRNA‐based TRIM47 targeting as a promising approach to improve HCC treatment outcomes.

## Graphical abstract

This study identifies TRIM47 as a key driver of liver cancer progression by promoting glycolysis through ubiquitin‐mediated degradation of the gluconeogenic enzyme FBP1. TRIM47 enhances glucose uptake, lactate and ATP production, and tumor growth and metastasis. Targeting TRIM47 restores metabolic balance and suppresses hepatocellular carcinoma progression, highlighting a promising metabolic therapeutic strategy.

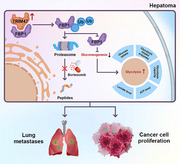

## 1 Introduction

Hepatocellular carcinoma (HCC) is the most common histological type of primary liver cancer and ranks as the sixth most common cancer and the third leading cause of cancer‐related death [1]. The asymptomatic property of early‐stage HCC often leads to late diagnoses, with most patients presenting at advanced stages when therapeutic options are limited, and prognosis is poor [2]. The increasing incidence and mortality rates of liver cancer highlight the urgent need for innovative therapeutic approaches [3]. A deeper understanding of the molecular mechanisms driving HCC progression is essential for identifying novel therapeutic targets.

Aberrant energy metabolism is a hallmark of cancer, enabling tumor cells to meet the increased energetic and biosynthetic demands of rapid proliferation [4, 5]. Glycolytic reprogramming, also known as the Warburg effect, is a prominent metabolic alteration wherein cancer cells preferentially utilize glycolysis for ATP production, even in the presence of adequate oxygen [6]. Current research indicates that the AMPK/AKT/mTOR signaling pathway plays a key role in linking cellular energy status with metabolic pathways, including glycolysis [7, 8]. AMPK, a key energy sensor and regulator of cellular energy homeostasis, is activated by phosphorylation at Thr172 under low‐energy conditions, which then inhibits mTOR activity [8, 9]. Conversely, aberrant activation of the AKT/mTOR pathway enhances glycolytic activity. mTOR functions as a central regulator of growth and metabolism, promoting glycolysis by inducing and regulating two critical transcription factors, HIF1α and c‐Myc [10]. Under normoxic conditions, c‐Myc, as a key driver of glycolysis, increases glucose uptake and glycolytic flux by upregulating the expression of GLUT1, HK2, PKM2, and LDHA to meet the energy requirements for maintaining the malignant phenotype of cancer cells [11]. This metabolic shift supports tumor growth and contributes to various malignant phenotypes, including proliferation, metastasis, angiogenesis, drug resistance, and immune evasion [8]. In HCC, dysregulated energy metabolism plays a pivotal role in disease progression [12, 13]. However, the specific molecular regulators orchestrating this metabolic reprogramming remain incompletely understood.

**FIGURE 1:**
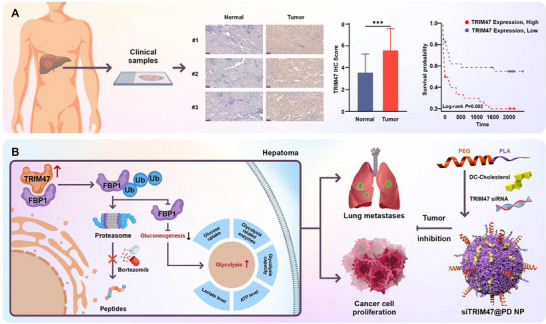
Mechanism of TRIM47 in HCC progression, and therapeutic potential of siTRIM47 nanoparticles (NPs). (A) TRIM47 is significantly upregulated in HCC tissues compared to normal tissues (*n* = 72, ^***^ *p* < 0.001. Data are expressed in mean ± SD), correlating with poorer patient survival (High = 36, Low = 36). (B) Schematic representation of TRIM47's role in HCC progression. TRIM47 promotes ubiquitin‐mediated degradation of FBP1, enhancing glycolysis, increasing lactic acid, adenosine triphosphate (ATP), and reactive oxygen species (ROS) production, which supports cancer cell proliferation and metastasis. Bortezomib inhibits TRIM47‐mediated FBP1 degradation, thereby reducing glycolytic activity. siTRIM47@PD NPs, consisting of PLA and DC‐Chol, target TRIM47, resulting in significant inhibition of tumor growth and metastasis, as demonstrated in an orthotopic HCC model.

Ubiquitination, an important post‐translational modification (PTM), plays a crucial role in cancer development and progression by regulating protein stability, localization, and activity [14, 15]. Emerging evidence suggests that ubiquitination significantly impacts aerobic glycolysis by modifying key enzymes or modulating signaling pathways, ultimately affecting cancer progression [14, 16, 17, 18]. In this study, we observed increased expression of tripartite motif‐containing protein 47 (TRIM47), the glycolysis‐associated ubiquitination‐related gene in patient‐derived HCC tissues (*p* < 0.001), and higher TRIM47 expression correlates with poorer patient outcomes (*p* = 0.002, Figure 1A). Additionally, weighted gene co‐expression network analysis (WGCNA) identified TRIM47 as being associated with glycolytic activity in HCC, suggesting its role in metabolic reprogramming. We therefore hypothesized that TRIM47 promotes glycolysis by interacting with key metabolic enzymes, although its precise role in HCC energy metabolism remains to be defined.

Given the crucial role of energy metabolism in HCC progression, targeting TRIM47‐mediated metabolic pathways presents a promising therapeutic approach. Small interfering RNA (siRNA) technologies offer the potential to specifically silence oncogenic genes; however, effective delivery remains a significant challenge due to siRNA instability and poor cellular uptake. Nanotechnology‐based delivery systems can enhance the stability and targeting capabilities of siRNA therapeutics [19]. In this context, poly lactic acid (PLA), a successful and widely utilized nanomaterial, has been extensively employed for drug delivery because of its high safety profile and approval by the United States Food and Drug Administration (FDA) [20, 21]. The use of PLA for targeted drug delivery is considered one of the safest nanomaterials for HCC treatment [22, 23]. However, nanomaterials, such as PLA, typically require the incorporation of cationic components to facilitate siRNA loading, thus forming nanocarriers with high transfection efficiency, biocompatibility, and biodegradability [24]. Incorporating cationic lipids such as 3β‐(N‐(N′, N′‐dimethylethylenediamine)‐carbamoyl) cholesterol (DC‐Chol) facilitates siRNA loading and cellular uptake [25].

This work aimed to elucidate the role of TRIM47 in HCC progression and evaluate its potential as a therapeutic target. Mechanistic investigations revealed that TRIM47 promotes HCC proliferation, metastasis, and glycolytic reprogramming through its interaction with fructose‐1,6‐bisphosphatase (FBP1), which drives enhanced glycolytic activity. We further demonstrated that FBP1 is essential for mediating the oncogenic effects of TRIM47, and bortezomib treatment effectively inhibited TRIM47‐mediated FBP1 degradation, reducing glycolysis and suppressing HCC progression. Additionally, we constructed a novel nanoplatform that combines PLA and DC‐Chol (PD) for siRNA delivery, aiming to evaluate the potential of siTRIM47@PD in HCC therapy. We demonstrated that intravenous administration of siTRIM47@PD significantly inhibited orthotopic HCC tumor growth in a mouse model, highlighting TRIM47 as a promising target for HCC therapy (Figure 1B). Collectively, these findings contribute to the understanding of HCC progression and support TRIM47 as a valuable target for the development of novel therapeutic strategies to improve patient outcomes.

## 2 Results

### 2.1 WGCNA Revealed That TRIM47, Associated with Glycolytic Activity, is a Key Gene Influencing the Progression of HCC

**FIGURE 2:**
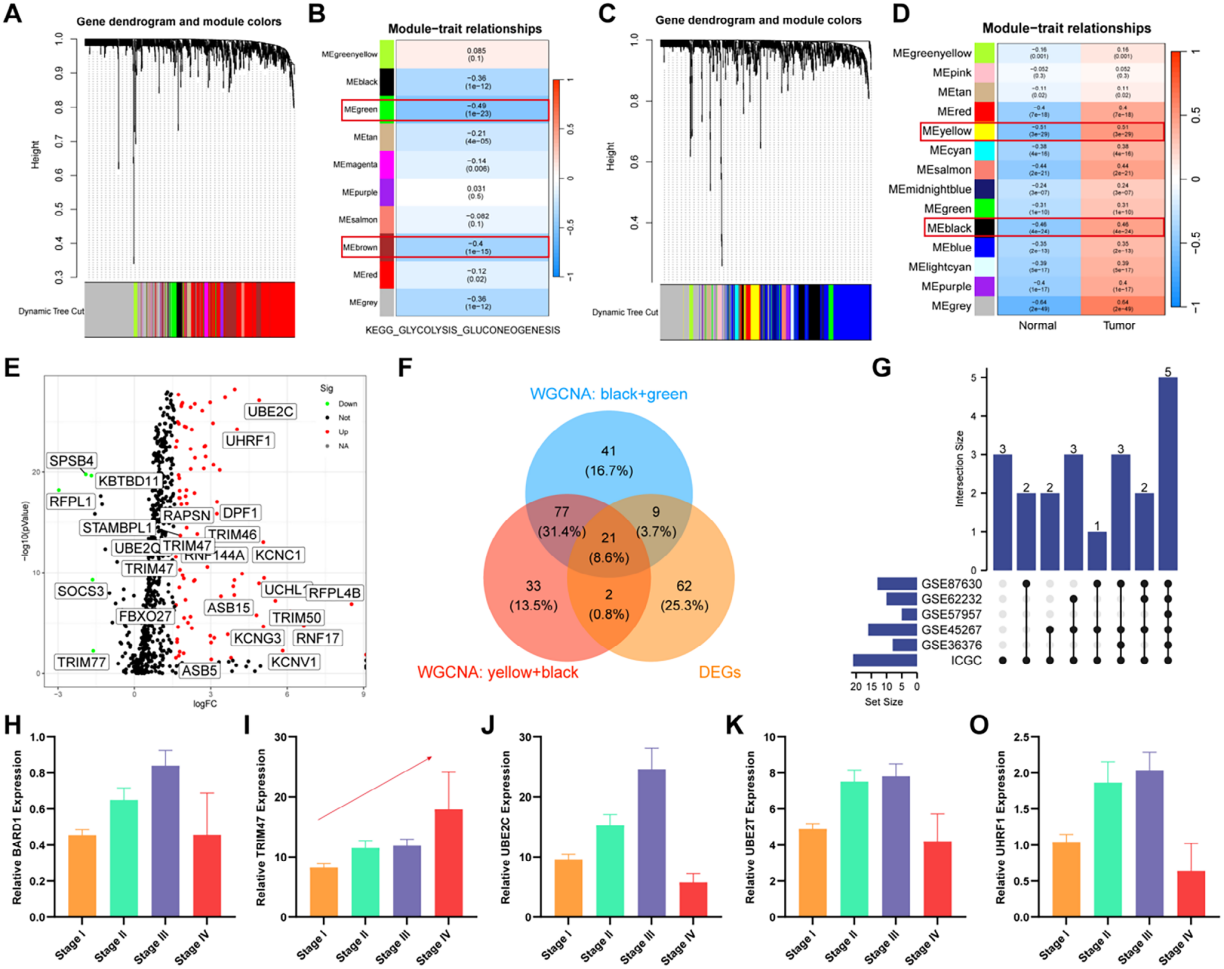
TRIM47, a sugar metabolism‐related protein, is a key PTM‐associated oncogene in HCC progression. (A) Cluster dendrogram of PTM associated with sugar metabolism identified via WGCNA. (B) Heatmap depicting the correlation between modules and sugar metabolism activity. (C) Cluster dendrogram of PTM associated with tumorigenesis identified via WGCNA. (D) Heatmap showing the correlations between modules and clinical traits. (E) Volcano plot illustrating the differential analysis of PTM. (F) Venn diagram depicting the overlap between key module genes (top 2) and DEGs. (G–O) Expression profiles of five key PTM‐related genes in different clinical stages.

To identify key PTM‐associated oncogenes that affect glycolytic activity in HCC and drive its progression, we initially scored glycolytic activity via the single‐sample Gene Set Enrichment Analysis (ssGSEA) algorithm in the Cancer Genome Atlas (TCGA)‐HCC and International Cancer Genome Consortium (ICGC)‐HCC datasets. We subsequently employed WGCNA to identify critical modules influencing glycolytic activity and tumor progression in HCC. Specifically, we identified seven modules associated with glycolytic activity (Figure 2A, B) and thirteen modules associated with tumor progression (Figure 2C, D). Additionally, we analyzed differentially expressed genes (DEGs) in tumors (Figure 2E). Based on these findings, we identified two key module genes affecting glycolytic activity (MEgreen and MRbrown) and tumor progression (MEyellow and MEblack), as well as common genes among the DEGs. Consequently, we identified a preliminary set of 21 potential DEGs that could impact HCC progression and glycolytic activity (Figure 2F). Furthermore, we validated the expression of these 21 genes via external databases, including the ICGC cohort, and Gene Expression Omnibus (GEO) cohort (GSE36376, GSE45267, GSE57957, GSE62232, and GSE87630). The results demonstrated stable differential expression of UBE2C, UBE2T, UHRF1, BARD1, and TRIM47 (Figure 2G). Notably, all five key genes exhibited prognostic value in HCC (Figure S1). Intriguingly, when the expression patterns of these key genes in different tumor stages were explored, only TRIM47 gradually increased with the tumor stage (Figure 2H–O). Consequently, we selected TRIM47 as the focus of our study.

To determine the significance of TRIM47 in HCC, we conducted an additional research analysis. The TRIM family, to which TRIM47 belongs, consists of 77 members in humans [26]. Initially, we performed a differential analysis of the 77 TRIM family genes in the TCGA‐HCC cohort (|log_2_Foldchange| > 1.585, *p* < 0.05) and generated a heatmap (Figure S2A) and a volcano plot (Figure S2B). Ultimately, we identified 23 DEGs, with one DEGs showing significantly decreased expression and the remaining 22 DEGs exhibiting significantly increased expression. We subsequently applied two machine learning algorithms, LASSO (Figure S2C) and random forest (Figure S2D), to further filter these 23 DEGs. This process resulted in 15 and 13 feature genes, respectively, and interestingly, nine TRIM family genes were identified as common feature genes (Figure S2E). By performing univariate Cox regression analysis for prognostic evaluation, we ultimately determined that three genes (TRIM16L, TRIM47, and TRIM59) significantly impact the prognosis of HCC patients (Figure S2F). To further validate these key genes, we expanded our analysis to include external HCC datasets, including the ICGC cohort, GSE36376, GSE45267, GSE57957, GSE62232, and GSE87630. Through differential analysis (|log_2_Foldchange| > 0.585, *p* < 0.05), we found that only TRIM47 exhibited significantly increased expression across all the datasets (Figure S3). Moreover, univariate Cox regression analysis of the key genes in the TCGA and ICGC cohorts revealed that TRIM47 alone was the crucial gene influencing the prognosis of HCC patients (Figure S2G,H). Furthermore, we analyzed the clinical relevance of TRIM47, as shown in Table S1, and found a correlation between TRIM47 expression and tumor TNM stage. Based on the comprehensive analysis, we consider TRIM47 a key PTM that influences glycolytic activity and progression in HCC.

### 2.2 TRIM47 Promotes the Proliferation, Migration, and Invasion Ability of HCC Cells

**FIGURE 3:**
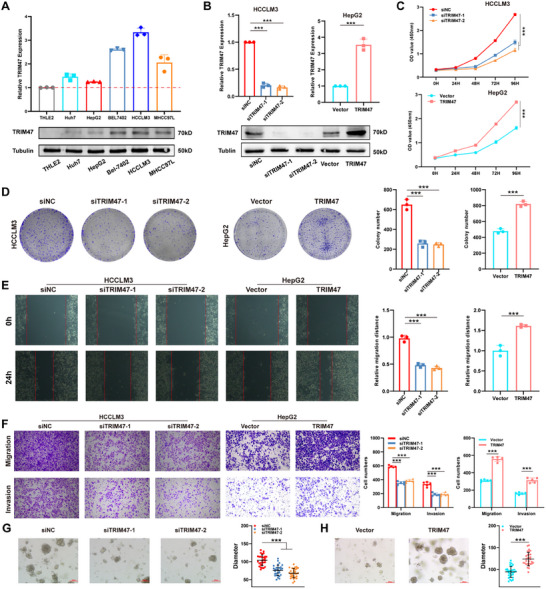
TRIM47 Promotes Proliferation, Migration, and Invasion Abilities of HCC Cells. (A) mRNA and protein expression levels of TRIM47 in THLE2, Huh7, HCCLM3, MHCC97L, HepG2, and Bel‐7402 cell lines (*n* = 3, Data are expressed in mean ± SD). (B) Efficiency of TRIM47 knockdown and overexpression at the mRNA and protein levels (*n* = 3, ^***^ *p* < 0.001. Data are expressed in mean ± SD). (C, D) CCK8 (*n* = 5, ^***^ *p* < 0.001. Data are expressed in mean ± SD) and colony formation assays (*n* = 3, ^***^ *p* < 0.001. Data are expressed in mean ± SD) were performed to assess the impact of TRIM47 expression on the proliferation ability of HCC cells. (E, F) Wound healing (*n* = 3, ^***^ *p* < 0.001. Data are expressed in mean ± SD) and Transwell assays (*n* = 5, ^***^ *p* < 0.001. Data are expressed in mean ± SD) were used to evaluate the influence of TRIM47 expression on the migration and invasion abilities of HCC cells. (G, H) Representative images and diameter statistics evaluating the effects of TRIM47 knockdown or overexpression on HCC PDOs viability (*n* = 30, ^***^ *p* < 0.001. Data are expressed in mean ± SD).

We subsequently investigated whether TRIM47 affects the malignant phenotype of HCC. We examined the expression of TRIM47 in the normal liver cell line THLE2 and five liver cancer cell lines (HepG2, Huh7, Bel‐7402, MCHH97L, and HCCLM3). The results revealed elevated mRNA and protein levels of TRIM47 in all five liver cancer cell lines (Figure 3A,B), with the highest expression observed in HCCLM3 cells and the lowest in HepG2 cells. Therefore, we selected HCCLM3 and HepG2 cells for in‐depth studies involving TRIM47 knockdown or overexpression. CCK8 and colony formation assays demonstrated that the knockdown of TRIM47 significantly impaired the proliferative capacity of HCCLM3 cells, whereas the overexpression of TRIM47 significantly enhanced the proliferative capacity of HepG2 cells (Figure 3C, D). Furthermore, we investigated the effects of TRIM47 on the migration and invasion abilities via wound healing and Transwell assays. The results revealed that the knockdown of TRIM47 significantly impaired the migration and invasion abilities of HCCLM3 cells, whereas the overexpression of TRIM47 significantly increased the migration and invasion abilities of HepG2 cells (Figure 3E,F). Finally, we established independent HCC patient‐derived organoids (PDOs) to validate the oncogenic role of TRIM47. We found that depletion of TRIM47 in HCC PDOs reduced growth (Figure 3G). Conversely, overexpression of TRIM47 significantly increased their growth (Figure 3H). Finally, the efficiency of TRIM47 knockdown was verified by Western blotting (WB) (Figure S4).

### 2.3 TRIM47 Facilitates Aerobic Glycolysis in HCC

**FIGURE 4:**
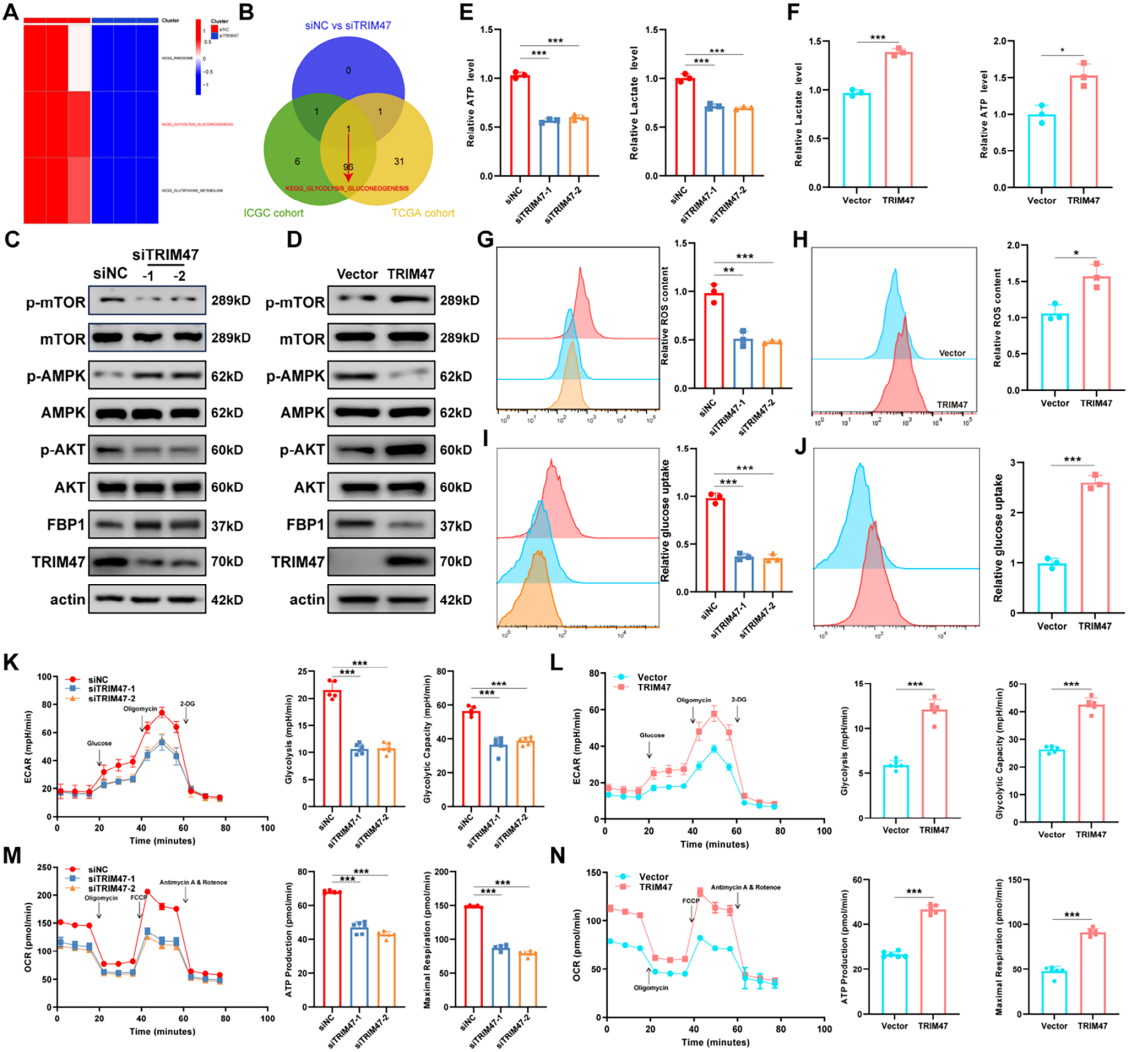
TRIM47 Promotes Glycolytic Activity in HCC Cells. (A) GSVA analysis of signaling pathways affected by TRIM47 knockdown. (B) Venn diagram displaying common downstream signaling pathways influenced by TRIM47 expression based on GSVA analysis of TCGA and ICGC data. (C,D) Effects of TRIM47 expression on the AMPK/AKT/mTOR signaling pathway. (E,F) Effects of TRIM47 expression on lactate production and ATP generation (*n* = 3, ^*^ *p* < 0.05, ^***^ *p* < 0.001. Data are expressed in mean ± SD). (G,H) Effects of TRIM47 expression on ROS generation. (I,J) Flow cytometry was used to detect the effect of TRIM47 expression on glucose absorption capacity. (K–N) Line graphs illustrate the effects of TRIM47 expression on ECAR and OCR. Differences in glycolysis, glycolytic capacity, ATP production, and maximal respiration are displayed as bar graphs.

Previous studies have suggested that the most likely PTM affecting glycolytic activity in HCC are mediated by TRIM47, thus implicating TRIM47 in promoting carcinogenesis. However, it remains unclear whether the oncogenic role of TRIM47 in HCC is primarily mediated through glycolytic activity. Therefore, we employed high‐throughput sequencing technology in HCC cells treated with siNC and siTRIM47 to investigate this phenomenon. Gene set variation analysis (GSVA) revealed significant suppression of the KEGG_RIBOSOME, KEGG_GLYCOLYSIS_GLUCONEOGENESIS, and KEGG_GLUTATHIONE_METABOLISM pathways in the siTRIM47 group (Figure 4A). Furthermore, based on median TRIM47 expression, we classified HCC patients from the TCGA and ICGC databases into high‐ and low‐expression groups and performed GSVA. Interestingly, we found that GLYCOLYSIS_GLUCONEOGENESIS was the only downstream pathway influenced by TRIM47 expression (Figure 4B). Combining these findings with those of previous analyses, we hypothesize that TRIM47 and glycolytic activity significantly influence HCC progression, with TRIM47 modulating HCC progression by regulating glycolytic activity. To validate this hypothesis, we first examined the effect of TRIM47 expression on the AMPK/AKT/mTOR signaling pathway. The results indicated that knockdown of TRIM47 led to increased phosphorylation of AMPK (p‐AMPK) and significantly reduced phosphorylation levels of AKT (p‐AKT) and mTOR (p‐mTOR) (Figure 4C). Conversely, overexpression of TRIM47 produced the opposite effects (Figure 4D). Additionally, we observed the impact of TRIM47 expression on the expression of glycolysis‐related regulatory genes (HIF‐1α, c‐Myc, GLUT1, PKM2, HK2, and LDHA). The results showed that knockdown of TRIM47 reduced the expression of glycolysis‐related regulatory genes (excluding HIF‐1α) (Figure S5A), while overexpression of TRIM47 led to an increase in their expression (Figure S5B). Meanwhile, based on previous studies [27], we treated cells overexpressing TRIM47 with a c‐Myc inhibitor (10058‐F4, MedChemExpress, USA). The results showed that inhibiting c‐Myc activity significantly eliminated the upregulation of glycolysis‐related genes (including GLUT1, PKM2, HK2, and LDHA) caused by TRIM47 overexpression (Figure S5C), indicating that the promotion of glycolysis by TRIM47 is largely dependent on the regulatory function of c‐Myc. Additionally, considering that aerobic glycolysis produces ATP with lactate as the main metabolic byproduct, we assessed the effect of TRIM47 expression on ATP and lactate production. The results demonstrated a significant reduction in ATP and lactate production upon TRIM47 knockdown, whereas overexpression of TRIM47 resulted in increased ATP and lactate production (Figure 4E,F). This preliminary evidence supports our hypothesis. Moreover, it has been reported that glycolysis can accelerate ROS accumulation [28]. Our results revealed a significant decrease in ROS accumulation upon TRIM47 knockdown, whereas TRIM47 overexpression led to a significant increase in ROS accumulation (Figure 4G,H).

Furthermore, we investigated the effect of TRIM47 on the cellular uptake of 2‐NBDG, which represents the glucose uptake capacity. Flow cytometry experiments revealed that glucose uptake capacity significantly decreased in HCCLM3 cells upon TRIM47 knockdown, whereas overexpression of TRIM47 in HepG2 cells led to a marked increase in glucose uptake capacity (Figure 4I,J). The same phenomenon was observed using confocal microscopy in immunofluorescence (IF) experiments (Figure S6). Finally, we measured the extracellular acidification rate (ECAR), which reflects overall glycolytic flux, and the oxygen consumption rate (OCR), an indicator of mitochondrial oxidative respiration. The ECAR results revealed a significant decrease in glycolysis and glycolytic activity upon TRIM47 knockdown, whereas the overexpression of TRIM47 resulted in a significant increase in glycolysis and glycolytic activity (Figure 4K,L). The OCR results revealed a significant decrease in ATP content and maximal respiratory capacity upon TRIM47 knockdown, whereas overexpression of TRIM47 led to a significant increase in ATP content and maximal respiratory capacity (Figure 4M,N).

### 2.4 Interaction between TRIM47 and FBP1 Proteins Mediates the Ubiquitination and Degradation of FBP1

**FIGURE 5:**
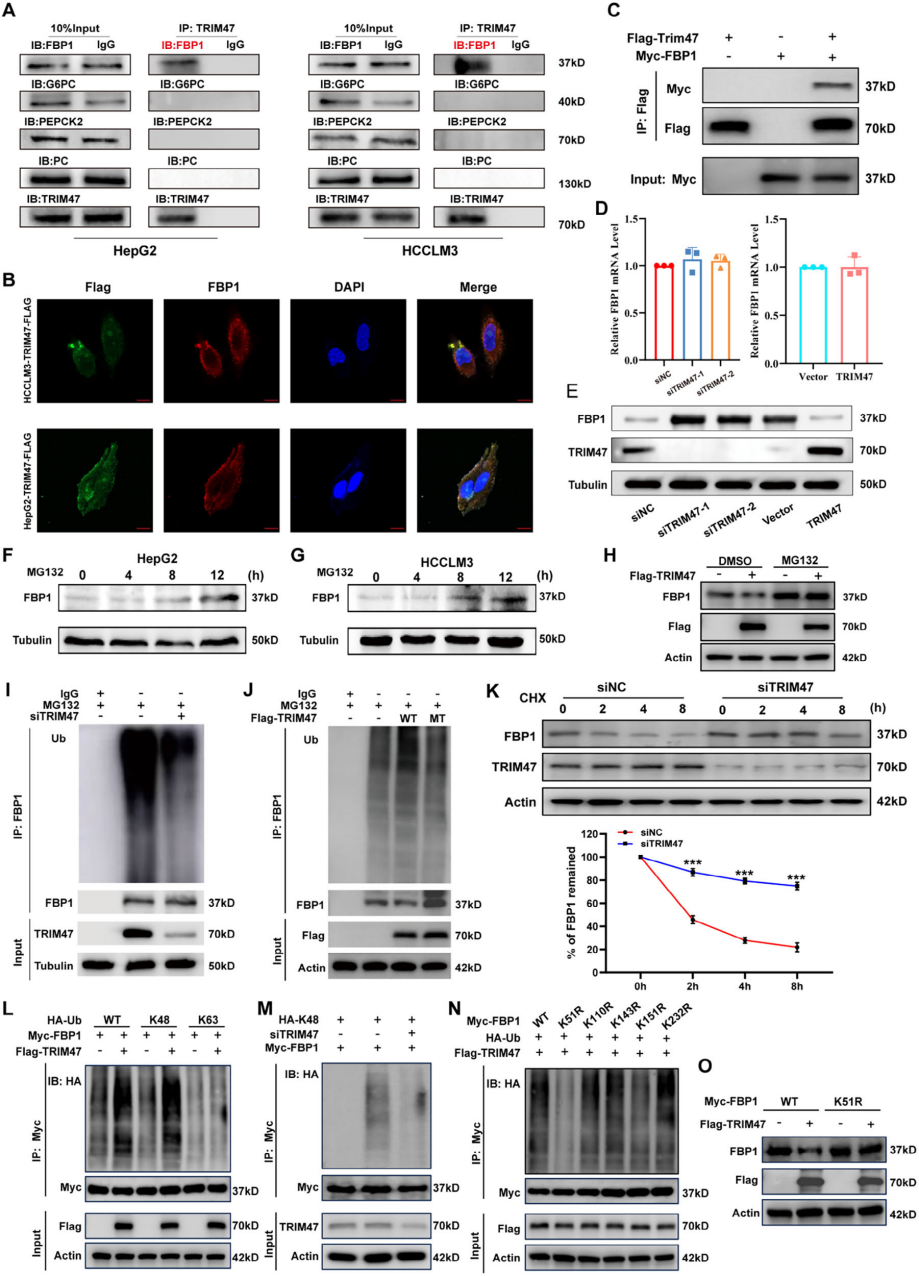
TRIM47 Interacts with FBP1 and Regulates its Protein Expression. (A) CoIP results of TRIM47 with key enzymes of the gluconeogenesis pathway in HepG2 and HCCLM3 cells. (B) Subcellular localisation of TRIM47 and FBP1 in HepG2 and HCCLM3 cells visualized by IF imaging. (C) CoIP results of ectopically expressed Flag‐TRIM47 and Myc‐FBP1 in HEK293T cells. (D, E) Impact of TRIM47 expression on the protein and mRNA levels of FBP1 (*n* = 3, Data are expressed in mean ± SD). (F,G) Changes in FBP1 protein levels in HepG2 and HCCLM3 cells upon MG132 treatment. (H) MG132 treatment abolished the effect of TRIM47 overexpression on FBP1 protein levels. (I,J) Under MG132 treatment, the impact of TRIM47 overexpression or knockdown on FBP1 ubiquitination levels was analyzed. (K) CHX chase assay demonstrated that TRIM47 knockdown affects the stability of FBP1 protein (*n* = 3, ^***^ *p* < 0.001. Data are expressed in mean ± SD). (L) Immunoprecipitation analysis of the types of polyubiquitination of FBP1 in HCC cells (with or without Flag‐TRIM47 lentiviral transfection) co‐transfected with Myc‐FBP1 and HA‐Ub‐K48 (K48‐linked ubiquitin only) or HA‐Ub‐K63 (K63‐linked ubiquitin only). (M) Immunoprecipitation analysis of FBP1 polyubiquitination in HCC cells (control and TRIM47 knockdown conditions) co‐transfected with Myc‐FBP1 and HA‐Ub‐K48. (N) CoIP between co‐transfected HA‐Ub and Myc‐FBP1 WT or lysine mutants to verify potential ubiquitination sites. (O) WB analysis of FBP1 protein levels in HCC cells (with or without Flag‐TRIM47 lentiviral transfection) transfected with WT or K51R Myc‐FBP1 plasmids.

Next, we focused on investigating how TRIM47 regulates glycolytic activity. We identified two key pieces of information that guided our selection of target proteins: 1) TRIM47 positively regulates glycolytic activity, and 2) TRIM47 functions as an E3 ubiquitin ligase that can ubiquitinate and degrade target proteins. Additionally, gluconeogenesis can antagonize the glycolytic pathway, and when one pathway is active, the other is suppressed [29]. Based on these findings, we hypothesized that TRIM47 affects the glycolytic pathway in HCC by directly regulating key enzymes involved in gluconeogenesis. Previous studies have identified key enzymes in the gluconeogenesis pathway, including G6PC, FBP1, PEPCK, and PC [12, 30, 31]. Therefore, we first explored whether TRIM47 interacts with these key enzymes. Coimmunoprecipitation (CoIP) results revealed that TRIM47 only interacts with the FBP1 protein in HCCLM3 and HepG2 cells and not with G6PC, PEPCK, or PC (Figure 5A). Furthermore, the IF results demonstrated that TRIM47 and FBP1 colocalized in the cytoplasm of the HCC cell lines HCCLM3 and HepG2 (Figure 5B), providing a physical basis for their interaction. Additionally, interaction proteomics (BioGrid) and molecular docking simulations (HDOCK) provided further evidence of a direct interaction between TRIM47 and FBP1 (Figure S7). To further verify their interaction within cells, we confirmed the interaction between endogenous TRIM47 and FBP1 through CoIP experiments (Figure S8). Importantly, we further validated this interaction in vitro using an in vitro binding assay (Figure 5C). We then explored the impact of TRIM47 on FBP1 expression and reported that knocking down TRIM47 increased FBP1 protein levels, whereas overexpressing TRIM47 decreased FBP1 protein levels (Figure 5D). Interestingly, the FBP1 mRNA level was not affected by TRIM47 expression (Figure 5E).

Given the regulatory effect of TRIM47 on FBP1 interaction, we treated cells with MG132, a proteasome inhibitor. We observed a time‐dependent increase in FBP1 expression in HCC cells after MG132 treatment (Figure 5F,G), and the regulatory effect of TRIM47 on FBP1 protein was abolished by MG132 (Figure 5H). Meanwhile, even in the presence of MG132, TRIM47 promoted the ubiquitination of FBP1 (Figure 5I,J). Next, we constructed a TRIM47 mutant plasmid (TRIM47 MT) in which the RING domain, essential for E3 ubiquitin ligase activity, was deleted. We found that TRIM47 had no effect on the ubiquitination level of FBP1 in the presence of MG132 (Figure 5J). These results suggest that TRIM47 regulates FBP1 protein expression primarily through ubiquitin‐mediated degradation. Finally, Cycloheximide (CHX) experiments revealed that the knockdown of TRIM47 retarded the degradation rate of FBP1 (Figure 5K). These results indicate that TRIM47 may shorten the half‐life of the FBP1 protein through its ubiquitination activity.

Subsequently, we further investigated the type of ubiquitination of FBP1 mediated by TRIM47. It has been reported that TRIM47 can regulate the ubiquitination level of protein substrates through either K48‐linked or K63‐linked ubiquitination [32, 33]. Our study discovered that in HCC cells, TRIM47 regulates the ubiquitination of protein substrates predominantly via K48 linkage rather than K63 linkage. Specifically, overexpression of TRIM47 significantly increased the K48‐linked ubiquitination level of FBP1 (Figure 5L), whereas knockdown of TRIM47 markedly decreased this K48‐linked ubiquitination (Figure 5M).

Similarly, we further identified the ubiquitination site within FBP1 that is influenced by TRIM47‐mediated K48‐linked ubiquitination. Previous studies had identified five potential ubiquitination sites on FBP1: K51, K110, K143, K151, and K232 [34]. Then, we constructed five lysine‐to‐arginine (K→R) mutants (K51R, K110R, K143R, K151R, and K232R) and performed CoIP assays to determine the functional ubiquitination sites. Our results indicated that only the K51R mutation significantly reduced FBP1 ubiquitination levels (Figure 5N). Furthermore, after the K51R mutation, TRIM47 overexpression no longer affected FBP1 protein levels (Figure 5O). In conclusion, these findings demonstrate that TRIM47 exerts its regulatory effect on FBP1 protein levels through K48‐linked ubiquitination at the K51 site on FBP1.

### 2.5 Involvement of FBP1 in the Biological Functions of TRIM47 in HCC

**FIGURE 6:**
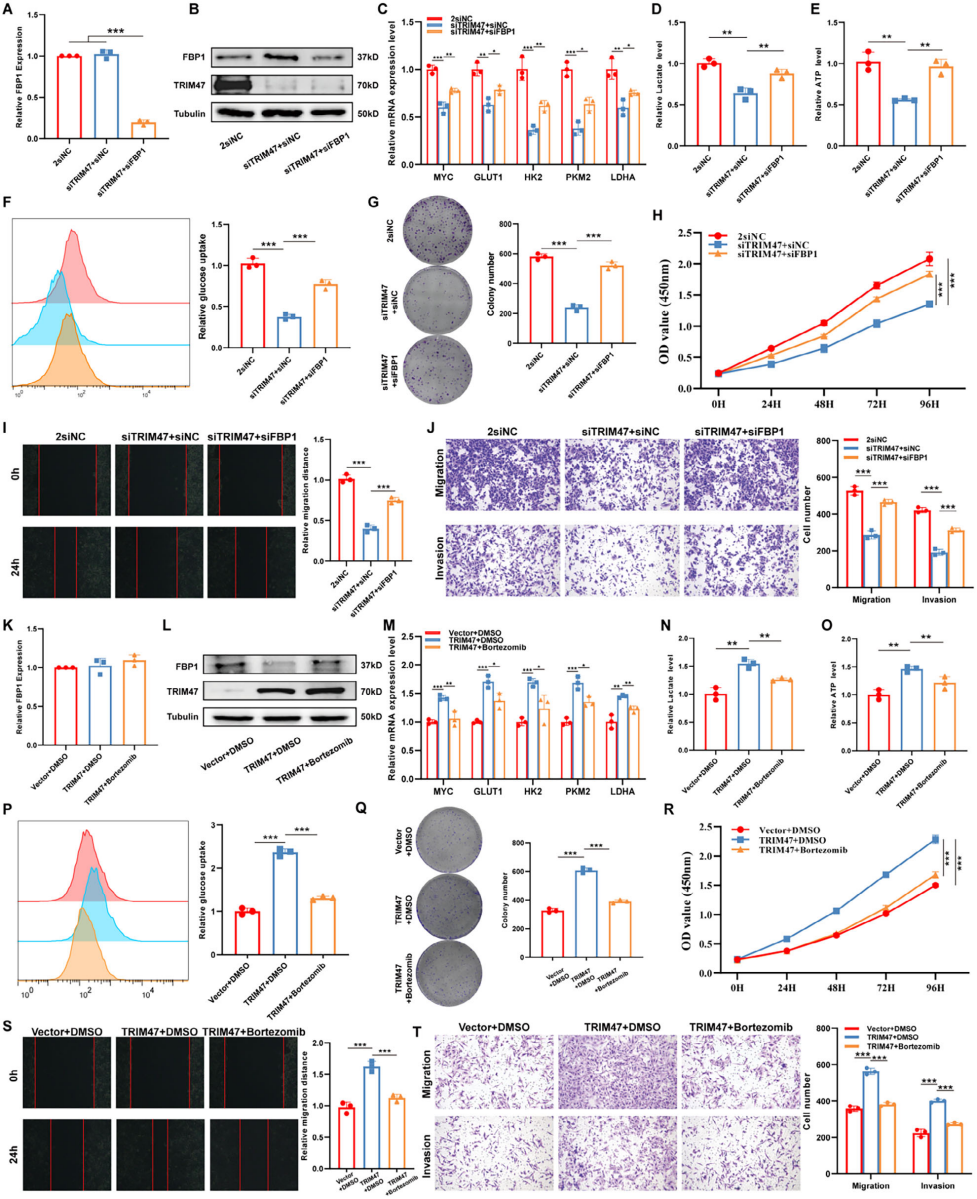
FBP1 Mediates the Oncogenic Role of TRIM47 in HCC. (A, B) Changes in the mRNA and protein levels of FBP1 after transfection with siTRIM47 and siFBP1 (*n* = 3, ^***^ *p* < 0.001. Data are expressed in mean ± SD). (C–F) Knockdown of FBP1 can rescue the reduction in key glycolysis‐related gene expression, lactate production, ATP generation, and glucose uptake caused by siTRIM47 (*n* = 3, ^*^ *p* < 0.05, ^**^ *p* < 0.01, ^***^ *p* < 0.001. Data are expressed in mean ± SD). (G–J) Cell function rescue experiments demonstrate that knocking down FBP1 can restore the decreased proliferation, migration, and invasion abilities of HCC cells induced by siTRIM47 (*n* = 3, ^***^ *p* < 0.001. Data are expressed in mean ± SD). (K, L) Changes in the mRNA and protein levels of FBP1 after transfection with siTRIM47 and bortezomib treatment. (M–P) Bortezomib treatment can inhibit the increase in key glycolysis‐related gene expression, lactate production, ATP generation, and glucose uptake induced by TRIM47 (*n* = 3, ^*^ *p* < 0.05, ^**^ *p* < 0.01, ^***^ *p* < 0.001. Data are expressed in mean ± SD). (Q–T) Bortezomib treatment can suppress the enhanced proliferation, migration, and invasion abilities of HCC cells induced by TRIM47 (*n* = 3, ^***^ *p* < 0.001. Data are expressed in mean ± SD).

Next, we investigated whether the biological function of TRIM47 in HCC depends on FBP1. First, we conducted rescue experiments by transfecting cells with siTRIM47, followed by either non‐transfection or siFBP1 plasmid transfection. We verified the success of the rescue experiments by assessing TRIM47 and FBP1 mRNA and protein levels through qPCR and WB (Figure 6A,B). We then examined whether FBP1 mediated TRIM47's regulation of glycolysis in HCC cells by analyzing changes in the expression of key glycolytic pathway genes (Figure 6C), lactate production (Figure 6D), ATP production (Figure 6E), and glucose uptake (Figure 6F). We found that the reduced glycolytic activity caused by TRIM47 suppression was significantly attenuated when FBP1 expression was also inhibited.

Finally, we evaluated whether FBP1 mediates TRIM47's oncogenic effects in HCC through CCK8 assays, colony formation assays, wound healing assays, and Transwell assays. The CCK8 assays and colony formation indicated that TRIM47 knockdown's inhibitory effect on HCC proliferation was significantly weakened when FBP1 was also knocked down (Figure 6G,H). The wound healing and Transwell assays showed that TRIM47 knockdown's inhibitory effect on HCC migration and invasion was significantly diminished when FBP1 was also knocked down (Figure 6I,J). These results suggest that the biological function of TRIM47 partially depends on FBP1.

To further confirm the functional dependency, we performed overexpression‐based rescue experiments in which TRIM47 was overexpressed alone or together with FBP1(Figure S9A). Consistent with the knockdown results, reconstitution of FBP1 expression markedly reversed the enhanced oncogenic phenotypes (Figure S9B–E) and glycolytic activity (Figure S9F–I) induced by TRIM47 overexpression, supporting that TRIM47 exerts its biological effects largely through regulating FBP1.

### 2.6 Bortezomib Disrupts TRIM47‐Mediated FBP1 Degradation and Partially Attenuates Its Oncogenic Effects

Bortezomib is a 26S proteasome inhibitor that has been shown to inhibit the malignant phenotype of HCC [35]. The mechanisms underlying the impact of bortezomib on HCC progression remain unclear. As a proteasome inhibitor, we investigated whether bortezomib could eliminate the degradation of FBP1 mediated by TRIM47. The results revealed that bortezomib abolished the inhibitory effect of TRIM47 overexpression on FBP1 protein levels but had no effect on FBP1 mRNA levels (Figure 6K,L).

In order to explore the impact of bortezomib on TRIM47‐mediated metabolic changes, we assessed lactate production and ATP levels in HCC cells. TRIM47 overexpression significantly enhanced the expression of key glycolytic pathway genes, lactate production, ATP production, and glucose uptake, indicating increased glycolytic activity. However, bortezomib treatment effectively suppressed the expression of key glycolysis‐related genes, lactate production, ATP generation, and glucose uptake, highlighting its role in inhibiting TRIM47‐driven metabolic reprogramming (Figure 6M–P). Furthermore, to determine the functional effects of bortezomib on TRIM47‐driven tumorigenic behaviors, we analyzed cell proliferation, migration, and invasion. Proliferation assays showed that TRIM47 overexpression significantly promoted HCC cell growth, while bortezomib treatment reduced this effect, as evidenced by CCK8 assay and colony formation assays (Figure 6Q,R). Additionally, TRIM47‐enhanced cell migration and invasion were markedly suppressed by bortezomib, as demonstrated by wound‐healing and Transwell assays (Figure 6S,T).

Finally, we evaluated whether TRIM47 regulates FBP1 expression and oncogenicity in HCC through its E3 ubiquitin ligase function by transfecting cells with either WT (Flag‐TRIM47 lentiviral plasmid) or MT (Flag‐TRIM47 mutant plasmid) plasmids. We observed that the TRIM47 WT plasmid significantly increased the level of ubiquitinated FBP1, whereas the TRIM47 MT plasmid failed to do so (Figure S10A), indicating that TRIM47's E3 ligase function is necessary for mediating FBP1 ubiquitination. Then, functional assays further demonstrated that the TRIM47 MT plasmid had no significant impact on HCC cell proliferation, migration, or invasion (Figures S10B–E), underscoring that the E3 ligase activity is critical for TRIM47's oncogenic effects. In metabolic assays, the TRIM47 WT plasmid enhanced the expression of key glycolytic pathway genes, lactate production, ATP production, and glucose uptake levels, but the TRIM47 MT plasmid showed no significant effect (Figures S10F–I), suggesting that TRIM47 regulates HCC energy metabolism specifically through its E3 ligase activity. Together, these findings demonstrate that TRIM47 promotes HCC glycolytic reprogramming and tumor progression by regulating FBP1 expression via the ubiquitin‐proteasome system.

### 2.7 TRIM47 Facilitates Tumor Growth and Metastasis In Vivo

**FIGURE 7:**
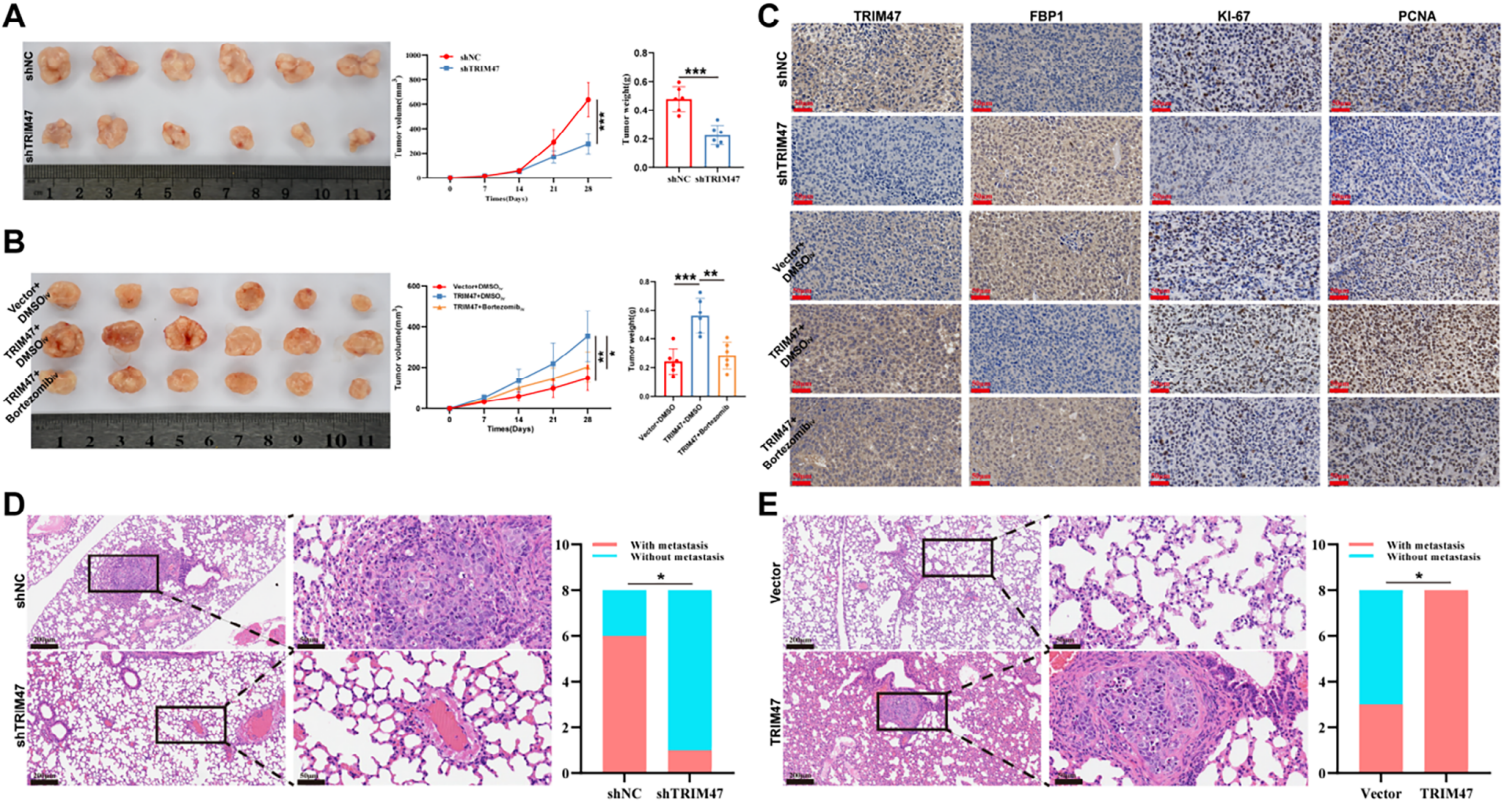
In Vivo Promoting Effect of TRIM47 on HCC Growth and Metastasis. (A) Representative images of xenograft tumors formed by HCCLM3 cells harbouring shNC or shTRIM47, along with comparisons of tumor growth rates and mass differences (*n* = 6, ^***^ *p* < 0.001. Data are expressed in mean ± SD). (B) Representative images of xenograft tumors formed by HepG2 cells harbouring vector or TRIM47, with or without bortezomib intervention, along with comparisons of tumor growth rates and mass differences (*n* = 6, ^*^ *p* < 0.05, ^**^ *p* < 0.01, ^***^ *p* < 0.001. Data are expressed in mean ± SD). (C) Representative IHC images of TRIM47, FBP1, KI‐67, and PCNA in xenograft tumors from mice subjected to different treatments. (D) Hematoxylin‐eosin (HE) staining images and metastasis ratios of lung metastatic lesions formed by HCCLM3 cells harbouring shNC or shTRIM47 (*n* = 8, ^*^ *p* < 0.05. Data are expressed in mean ± SD). (E) HE staining images and metastasis ratios of lung metastatic lesions formed by HepG2 cells harbouring vector or TRIM47 (*n* = 8, ^*^ *p* < 0.05. Data are expressed in mean ± SD).

Based on preliminary cellular experiments, we investigated the impact of TRIM47 expression on tumor growth in a subcutaneous xenograft mouse model. The results revealed a significant decrease in tumor volume and size in the HCCLM3 shTRIM47 group compared with the HCCLM3 shNC group (Figure 7A). Conversely, compared with the HepG2 vector group, the HepG2 TRIM47 group presented significant increases and were significantly reduced after bortezomib treatment in tumor volume and size (Figure 7B). We subsequently performed immunohistochemical (IHC) analysis of TRIM47, FBP1, KI‐67, proliferating cell nuclear antigen (PCNA), and c‐Myc in the tumor tissues (Figure 7C,D). On the one hand, the IHC results confirmed the effectiveness of TRIM47 knockdown and overexpression in promoting tumor formation. The altered expression of FBP1 indicated its regulation by TRIM47 in vivo. The IHC results for KI‐67 and PCNA suggested that TRIM47 knockdown inhibited tumor cell proliferation in vivo, while TRIM47 overexpression promoted proliferation. Finally, the IHC results for c‐Myc indicated that TRIM47 could influence the glycolytic capacity of tumor cells in a subcutaneous xenograft mouse model. Furthermore, we assessed the impact of TRIM47 on tumor metastasis via a lung metastasis model, and the results revealed a significant reduction in the number of lung metastases in the HCCLM3 shTRIM47 group compared with the HCCLM3 shNC group (Figure 7E).

### 2.8 Partial Inhibition of HCC Growth by PLA‐Encapsulated TRIM47 siRNA

**FIGURE 8:**
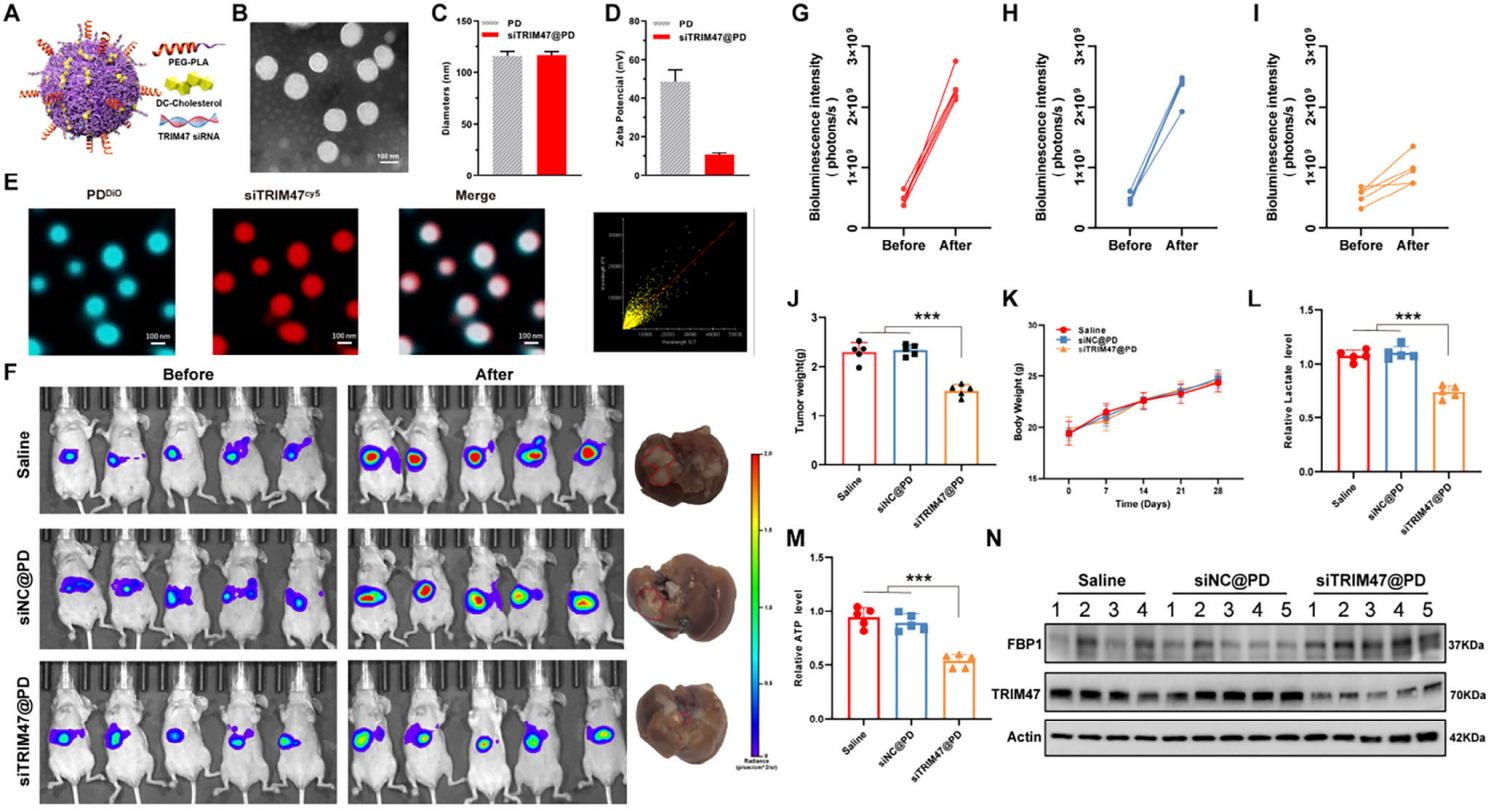
In Vivo Therapeutic Efficacy of siTRIM47@PD NPs in Orthotopic Liver Cancer Mice. (A) Schematic diagram of the NPs structure. (B) Transmission electron microscopy image of siTRIM47@PD NPs. (C) Particle size distribution of PD and siTRIM47@PD. (D) Zeta potential of PD and siTRIM47@PD. (E) Statistical analysis of PD^DIO^ on siTRIM47@PD and the corresponding fluorescence signal of siTRIM47^Cy5^ via the Pearson correlation test, with a correlation coefficient of 0.8651. (F) Bioluminescence and representative gross images of the liver in the saline, siNC@PD, and siTRIM47@PD groups. (G–I) Compared with those in the saline and siNC@PD groups, the fluorescence intensity in the siTRIM47@PD group was significantly lower. (J) Compared with those in the saline and siNC@PD groups, the liver tumor weight in the siTRIM47@PD group was significantly lower (*n* = 5, ^***^ *p* < 0.001. Data are expressed in mean ± SD). (K) Mouse weight change curve. (L,M) Lactate levels and ATP levels in liver tumor tissues of the saline group, siNC@PD group, and siTRIM47@PD group. (N) WB experiments explored the expression of TRIM47 and FBP1 proteins in liver tumor tissues of the saline group, siNC@PD group, and siTRIM47@PD group (*n* = 5, ^***^ *p* < 0.001. Data are expressed in mean ± SD).

To investigate the potential of TRIM47 as a therapeutic target for HCC, we examined the in vivo anticancer activity of siTRIM47 encapsulated in PLA NPs in an orthotopic liver cancer model. A schematic representation of the PLA is shown in Figure 8A, and transmission electron microscopy revealed that the PLA NPs were spherical with a diameter of approximately 120 nm (Figure 8B). Upon siRNA loading, there was a slight increase in the volume of the NPs (Figure 8C), whereas the zeta potential significantly decreased (Figure 8D). Furthermore, fluorescence microscopy demonstrated effective uptake of the NPs by tumor cells (Figure 8E). In addition, in vitro release studies showed that siTRIM47@PD NPs exhibited a pH‐responsive release profile, with accelerated siRNA release under mildly acidic conditions (pH 5.0 and pH 6.8) compared with physiological pH 7.4, indicating their suitability for endosomal escape and effective intracellular delivery (Figure S11). We then evaluated the biological toxicity of the NPs. HE staining showed that the siTRIM47@PD NPs did not cause obvious inflammation or damage to the major organs (heart, liver, spleen, lungs, and kidneys) of nude mice (Figure S12). In addition, we monitored various biochemical parameters in the blood of the mice, and the results indicated the absence of biological toxicity from the NPs formulation (Figure S13). Finally, pharmacokinetic studies showed that the concentration duration of siTRIM47@PD NPs in mice was significantly prolonged compared with siTRIM47 (Figure S14).

We injected the HCC cell line HCCLM3 into the livers of 4‐week‐old BALB/c nude mice, and after 14 days, the mice displayed similar tumor bioluminescence intensities. These mice were treated with either a phosphate‐buffered saline/NPs formulation siRNA@PD every three days via tail vein injection for a total of four times, and the bioluminescence intensity of the tumors was monitored continuously for four weeks. By bioluminescence, it was found that the siNC@PD group did not show any therapeutic effect compared with the Saline group, whereas the siTRIM47@PD group presented significantly suppressed tumor growth (Figure 8F–I). We supported this view by comparing the changes in liver tumor weight among the groups (Figure 8J). Moreover, there was no significant difference in body weight among the groups during the treatment period (Figure 8K), which also supports the biosafety of the NPs. In the in vivo metabolic assessment, we found that siTRIM47@PD NPs significantly reduced lactate and ATP levels in liver tissues (Figure 8L, M). Finally, WB results indicated that siTRIM47@PD NPs significantly suppressed the expression of TRIM47 and promoted the expression of FBP1 in the tissues (Figure 8N). These results indicate that siTRIM47@PD NPs effectively inhibit TRIM47 expression, regulate FBP1 and glycolytic activity, thereby suppressing tumor growth, with excellent biosafety.

In conclusion, our findings indicate that TRIM47 can promote glycolysis activity and HCC progression by ubiquitinating and degrading FBP1. This provides a novel approach for treating HCC using siTRIM47.

## 3 Discussion

Metabolic reprogramming is a crucial hallmark of cancer, with aerobic glycolysis (the Warburg effect) being one of the most prominent examples [36]. Aerobic glycolysis was initially observed in HCC [37]. While aerobic glycolysis generally implies a less efficient energy metabolism process, it enables rapid energy production to meet the demands of tumor cells [38]. Consequently, the regulation of glycolysis during the occurrence and progression of HCC has garnered significant attention. However, the role of ubiquitin family members in modulating glycolytic activity during HCC development remains unknown. Our study revealed that TRIM47, a protein associated with glycolytic activity, enhances aerobic glycolysis and promotes HCC progression.

Clinical studies have shown significant overexpression of TRIM47 in HCC, and high levels of TRIM47 expression are associated with poor prognosis. Functionally, TRIM47 significantly promotes HCC cell proliferation, migration, invasion, tumor growth, and metastasis. Previous research has indicated that TRIM47 can influence disease progression through the ubiquitin‐mediated degradation of target proteins [33, 39]. In this study, we employed GSVA to identify the potential impact of TRIM47 on HCC progression through the glycolysis pathway. Importantly, through a series of experiments, we demonstrated that TRIM47 physically interacts with FBP1 and promotes glycolytic activity by ubiquitinating and degrading the FBP1 protein. Specifically, TRIM47 mediates K48‐linked polyubiquitination at the K51 site of FBP1, leading to its proteasomal degradation. Previous studies have shown that the downregulation of FBP1 in HCC and the inhibition of glycolysis through FBP1 suppression can impede HCC progression [40]. Furthermore, our research confirmed that the oncogenic role of TRIM47 in vitro and in vivo is mediated by FBP1. A recent study reported that TRIM47 promotes HCC progression by stabilizing SNAI1 and enhancing metastasis, highlighting its role in regulating protein homeostasis through ubiquitination [41]. While that study focused on epithelial–mesenchymal transition and invasion pathways, our findings expand the understanding of TRIM47's oncogenic mechanisms by revealing, for the first time, that TRIM47 promotes HCC progression by enhancing glycolysis through the ubiquitin‐mediated degradation of FBP1. This underscores a novel link between TRIM47 and metabolic reprogramming, providing fresh insights into how TRIM47 drives tumor growth beyond its previously known functions. To our knowledge, this is the first study to demonstrate that TRIM47 promotes HCC progression by enhancing glycolysis through its interaction with FBP1.

Bortezomib, a 26S proteasome inhibitor, has been approved by the FDA for treating multiple myeloma and mantle cell lymphoma [42]. Furthermore, bortezomib has shown potential in inhibiting HCC progression, indicating its therapeutic potential for HCC treatment [43, 44]. In this study, we discovered that bortezomib markedly reduces FBP1 ubiquitination and degradation mediated by TRIM47, thereby inhibiting HCC progression and glycolytic activity. Importantly, we developed a PD NPs platform carrying TRIM47 siRNA to explore the possibility of targeting TRIM47 as a therapy for HCC. The PD NPs platform improves the stability and tumor targeting of siRNA. Given that the liver is the primary organ for drug metabolism, significant accumulation of NPs can be achieved in the liver [45]. To the best of our knowledge, this is the first study to use a NPs platform to target TRIM family members for HCC treatment. Our results demonstrate that siTRIM47@PD NPs therapy effectively inhibits the growth of orthotopic liver cancer. Importantly, the siTRIM47@PD NPs complex exhibited no organ toxicity in vivo. These findings suggest the feasibility and safety of siTRIM47@PD NPs for HCC treatment.

In summary, our research demonstrated the overexpression of TRIM47 in HCC and its association with poor prognosis. In vitro and in vivo experiments revealed the oncogenic role of TRIM47 in HCC. Mechanistically, TRIM47 enhances glycolytic activity by interacting with FBP1 and promoting its K48‐linked ubiquitination at the K51 site, leading to FBP1 degradation. Moreover, bortezomib effectively inhibits the ubiquitination and degradation of FBP1 mediated by TRIM47, suppressing HCC progression. Finally, we developed an NPs‐based therapeutic platform encapsulating TRIM47, which significantly inhibits the growth of HCC without causing organ toxicity. These findings provide promising gene therapy targets for the clinical treatment of HCC. These findings have significant clinical implications for a deeper understanding of the pathogenesis of HCC and the development of more effective treatment strategies.

## 4 Materials and Methods

### 4.1 Clinical Samples

Approval was obtained from the Ethics Committee of the First Affiliated Hospital of Anhui Medical University (LLSC‐2022424). Informed consent was obtained from all patients involved in the study. The study was conducted in accordance with the Declaration of Helsinki. All patients participating in the study have given informed consent. The inclusion criteria for HCC cohorts were: (1) initial diagnosis of hepatocellular carcinoma confirmed by histopathological examination; (2) no prior anticancer treatments (e.g., chemotherapy, radiotherapy, or targeted therapy) before surgery; (3) availability of complete clinical and follow‐up data; and (4) provision of written informed consent. The exclusion criteria were: (1) presence of other synchronous malignancies; (2) history of other malignancies; (3) severe comorbidities (e.g., heart failure, renal insufficiency); (4) poor‐quality RNA or protein extracts from the collected tissue samples.

The study consisted of two cohorts. Cohort 1 included 72 HCC patients who underwent surgical procedures at the First Affiliated Hospital of Anhui Medical University between January 2014 and April 2015. Cancer tissues and adjacent noncancerous tissues were collected from these patients. The clinical and prognostic data of the 72 HCC patients were also collected and preserved in paraffin blocks for long‐term storage. These samples were utilized for differential analysis of TRIM47 at the protein level, prognosis analysis, and clinicopathological characterization of TRIM47 in HCC. Cohort 2 included 40 HCC patients who underwent surgical procedures at the First Affiliated Hospital of Anhui Medical University between January 2022 and April 2023. Cancer and adjacent noncancerous tissues were collected and stored as frozen samples at −80°C. These samples were used for differential analysis of TRIM47 at the mRNA level.

### 4.2 Cell Culture, Transient Transfection, Lentiviral Infection, and Bortezomib Intervention

All cell lines (THLE2, HCCLM3, HepG2, MCHH97L, Bel‐7402, and Huh7) were generously provided by Dr. Dai WQ [46]. The cells were cultured in DMEM or RPMI‐1640 medium supplemented with 10% foetal bovine serum and 1% penicillin‐streptomycin. Transient transfection was performed according to previously established protocols [47]. The shNC and shTRIM47 lentiviral plasmids were purchased from Tsingke Biotech (Beijing, China). The vector and TRIM47‐Flag lentiviral supernatants were constructed by GenePharma Biotech (Shanghai, China). After viral infection of the target cells for 48 h, the cells were cultured in a medium containing 2 µg/mL puromycin for 36 h for selection. The transfection efficiency was validated via qPCR and WB. Additionally, for the bortezomib (MCE, China) cell intervention experiments, the cells were cultured in a medium supplemented with 20 µm bortezomib for 24 h and then subjected to relevant cell experiments after the medium was replaced with a complete culture medium. Detailed information regarding the siRNA and shRNA sequences information used in this study can be found in Table S2.

### 4.3 Cell Functional Experiments

CCK‐8, scratch, and Transwell assays were performed as described in our previous studies [47, 48]. For the colony formation assay, 1000 cells were seeded in a 6‐well plate (Corning, USA) and cultured for 12 days. The experiment was terminated, and the cells were fixed with 4% paraformaldehyde (Labgic, China) and stained with crystal violet (Beyotime Biotechnology, China). The colonies were counted via image analysis software.

### 4.4 IHC and IF

The IHC protocol was conducted following previous studies [49]. The anti‐TRIM47 antibody used in this study was diluted at a 1:200 ratio. Two experienced pathologists independently analyzed all the IHC staining and performed semiquantitative scoring.

The scoring system quantified the percentage of stained cells as follows: 4 (76%–100%), 3 (26%–75%), 2 (6%–25%), 1 (1%–5%), and 0 (0%). The staining intensity was scored as follows: negative (0), weakly positive (1), moderately positive (2), or strongly positive (3). The IHC score was determined by multiplying the percentage of stained cells by the staining intensity score for TRIM47. An IHC score greater than 6 was defined as high expression, whereas a score less than 6 was considered low.

For the IF experiments, cells were seeded in confocal dishes at a density of approximately 30% to initiate the experiments. The cells were fixed with 4% paraformaldehyde for 20 min, permeabilized with 0.5% Triton X‐100 (Meilunbio, China) for 10 min, and blocked with blocking serum (working solution) by shaking on a shaker for 15 min. The appropriate primary antibodies, Flag and FBP1 (Proteintech, China, 1:100), were added, and the samples were incubated overnight. After a 30 min rewarming period, the corresponding fluorescent secondary antibodies (1:100) were added, and the samples were incubated in the dark for 60 min. Finally, a DAPI working solution (1:100) was added, and the samples were incubated in the dark for 5 min. The images were captured via a confocal microscope.

### 4.5 qRT‐PCR, CoIP, and WB

RNA extraction and qRT‐PCR were performed according to previous studies [50]. Briefly, total RNA was extracted from frozen tissues or cells via Trizol (Thermo Fisher Scientific, USA). Synthesis and amplification of cDNA were carried out via the PrimeScript RT Master Mix (Takara Bio, Japan). TB‐Green qPCR (Takara Bio, Japan) was used for the quantitative analysis of TRIM47, with GAPDH as the internal control. Detailed information regarding the primer sequences used in this study can be found in Table S3.

For the CoIP experiment, cells (greater than 1x10^7) were lysed with 1 mL of lysis buffer (IP: PMSF = 99:1) for 40 min. A portion of the lysate was saved for the Input group, while the remaining lysate was incubated overnight at 4°C with the appropriate primary antibody (100:1) on a rocking shaker. The next day, protein A/G agarose beads (Santa Cruz, USA, 5:1) were added and incubated for 2–4 h, followed by three washes with PBST. The input group samples were mixed with 5X loading buffer, heated at 100°C in a metal heating block for 10 min, and stored at −80°C for subsequent WB experiments.

For WB experiments, the cells were lysed with lysis buffer for 40 min, and the supernatant was collected after centrifugation at 12 000 rpm for 15 min. The supernatant was mixed with 5X loading buffer and heated at 100°C in a metal heating block for 10 min. Equal amounts of total protein were subjected to 7.5% or 10% SDS‒PAGE and then transferred to a PVDF membrane (Merck, USA). After the membranes were blocked with 5% skim milk, they were incubated overnight at 4°C with specific primary antibodies. The membrane was subsequently incubated with the corresponding secondary antibodies for 2 h, followed by detection using an ECL substrate. Detailed information regarding the antibody information used in this study can be found in Table S4.

The Myc‐FBP1 mutants mentioned in the article were generated by the Site‐Directed Mutagenesis Kit (R401; Takara Bio, Tokyo, Japan).

When protein ubiquitination was determined, cells were harvested in the RIPA lysis buffer containing N‐ethylmaleimide (10 mm). The immunoprecipitates were washed three times with the high‐salt (500 mm NaCl) washing buffer and twice with the normal (150 mm NaCl) washing buffer, and then were subjected to immunoblotting analysis as above.

### 4.6 In Vitro Binding Assay

HEK293T cells were transfected with the constructs. Flag‐Trim47 was immunoprecipitated and then eluted with the Flag peptides, respectively. Myc‐FBP1 was pulled down by the Myc antibody from cells transfected with Myc‐FBP1. Then Flag‐Trim47 eluates were mixed and vibrated with the Myc‐FBP1 immunoprecipitates at 4°C. The products of this reaction were separated by SDS‐PAGE and analyzed by immunoblotting using the anti‐Flag or anti‐Myc antibody.

### 4.7 Glucose Uptake, Lactate Production, ATP Generation, and ROS Generation

To assess glucose uptake capacity, we monitored glucose uptake in HCCLM3 and HepG2 cells using flow cytometry and confocal microscopy. For flow cytometry, experimental cells were grown to approximately 60% confluence in 6‐well plates and then switched to glucose‐free medium overnight. Cells were then incubated in glucose‐free DMEM containing 80 µm 2‐NBDG (Thermo Fisher Scientific, USA) for 2 h. After collecting the cells, they were washed three times with PBS, measured using a FACS flow cytometer (BD Biosciences, USA), and the data were analyzed with FlowJo software. Meanwhile, experimental cells were seeded in confocal dishes (Thermo Fisher Scientific, USA) at a density of 20%–30% and cultured overnight in a sugar‐free medium. The cells were subsequently incubated in glucose‐free DMEM containing 20 µm 2‐NBDG (Thermo Fisher Scientific, USA) for 2 h. After being washed with PBS, the cells were fixed with 4% paraformaldehyde, and a DAPI working solution (Sangon Biotech, China) was added to stain the cell nuclei for 5 min. The stained sections were visualized via a confocal microscope (Zeiss, Germany).

For intracellular lactate and ATP production measurement: When the density of the experimental cells reached approximately 80%, the medium was replaced, and after 24 h, the supernatant was collected to measure lactate levels, while the ATP levels in the cells were measured. The experimental procedures were performed according to the manufacturer's instructions (Nanjing Jiancheng, China).

For lactate and ATP production measurement in tissues: Equal weights of liver tissues from each experimental group were homogenized with 9 volumes of saline on ice. The homogenate was centrifuged at 2500 rpm for 10 min, and the supernatant was used for lactate and ATP level measurements, following the manufacturer's instructions (Nanjing Jiancheng Bioengineering Institute, China).

For ROS detection, cells in good condition were collected and incubated with DCFH‐DA working solution (Beyotime Biotechnology, China) at room temperature for 30 min. The average fluorescence intensity of the cells, which represents the intracellular ROS content, was measured via FACS flow cytometry, with excitation at a wavelength of 488 nm.

### 4.8 ECAR and OCR Determination

For ECAR and OCR measurements, 10 000 cells were seeded in Seahorse XF Cell Culture Microplates overnight for subsequent experiments. A Seahorse XF Glycolysis Stress Test Kit and Seahorse XF Cell Mito Stress Test Kit (Agilent Technologies, USA) were used to measure the cell ECAR and OCR, respectively, via a Seahorse XF 96 Extracellular Flux Analyser (Agilent Technologies, USA).

### 4.9 Establishment and Cultivation of HCC PDOs

The establishment and culture of HCC organoids were carried out according to previously published protocols [51]. In brief, liver cancer samples derived from HCC patients, supplied by the First Affiliated Hospital of Anhui Medical University, were used to generate PDOs. Fresh HCC tissue was washed with primary buffer, debris was removed, and the tissue was further cut into approximately 1 mm fragments. These tissue pieces were then digested with primary tissue digestion solution for 15–25 min. When an abundance of cell clusters or single cells smaller than 70 µm was observed, digestion was halted, and the tissue slurry was filtered through a 100 µm cell strainer. The filtrate was centrifuged at 200 rpm for 5 min to collect the tissue cell pellet. The pellet was then mixed with an equal volume of Matrigel to form a 3D culture scaffold. After solidification, the organoids were cultured in organoid medium (Lzbiotech, China) and incubated at 37°C in a CO_2_ incubator.

### 4.10 Nude Mouse Subcutaneous Tumor Model and Lung Metastasis Model

Nude Mouse Subcutaneous Tumor Model: Four‐week‐old male nude mice (BALB/c‐nu, GemPharmatech, China) were subcutaneously injected with a suitable amount of stable clone cells (shNC/shTRIM47: 3 × 10^6^, 50 µL; Vector/TRIM47: 2 × 10^6^, 50 µL). Mice were divided into groups of six, and after cell injection, subcutaneous tumor size was observed and measured weekly. Four weeks later, the mice were euthanized, subcutaneous tumors were surgically removed, tumor weight was recorded, and samples were stored long‐term in paraffin.

Lung Metastasis Model: Experimental cells were injected into the tail vein of four‐week‐old male nude mice at a dose of 2 × 10^6^ (100 µL). Six weeks later, the mice were euthanized, lung tissues were surgically removed, and stored long‐term in paraffin.

All animal experiments were approved by the Ethics Committee of the First Affiliated Hospital of Anhui Medical University (LLSC‐20231225). All animals in this study were handled in accordance with the guidelines of Anhui Medical University and approved by the Animal Care and Use Ethics Committee of Anhui Medical University.

### 4.11 Preparation and Characterization of siRNA@PD

PLA, DC‐cholesterol, and PLA‐PEG powders were dissolved in dichloromethane at a mass ratio of 10:5:10. The mixture was then emulsified by adding it into an aqueous phase containing sodium cholate as a surfactant and subjected to ultrasonication. After vigorous stirring for 24 h, the dichloromethane was evaporated, followed by centrifugation at 15 000 rpm for 30 min to discard the supernatant and collect the NPs. The obtained PD NPs were washed with deionized water and centrifuged at 15 000 rpm for 30 min to remove impurities. Finally, the PD NPs were mixed with siRNAs (siNC and siTRIM47) at a 30:1 mass ratio and incubated for 2 h to obtain siNC@PD and siTRIM47@PD NPs. The zeta potential and particle size of the PD and siTRIM47@PD NPs were measured via a Zetasizer Nano ZS instrument (Malvern, UK). The NPs were imaged via a Delta Vision OMX structured illumination super‐resolution fluorescence microscope (Nikon, Japan) and a transmission electron microscope (Thermo Fisher Scientific, China).

### 4.12 In Vivo Toxicity and Antitumor Efficacy of the siRNA@PD NPs and Pharmacokinetic Evaluation

Toxicity Evaluation: On days 0, 3, and 6, 6‐week‐old male BALB/c nude mice were injected with saline and siRNA@PD NPs (1 mg/kg) through the tail vein (*n* = 5). The experiment was terminated on day 10 for safety assessment. On the one hand, the important organs of nude mice (heart, liver, spleen, lung, and kidney) were taken, embedded in paraffin, and HE staining was performed to observe the pathological changes of each tissue to evaluate whether the tissue had inflammation or damage. On the other hand, venous blood was taken from the mice through the eyeballs, and the health status of the mice was reflected by detecting conventional biochemical indicators.

Anti‐tumor effect evaluation: male athymic nude mice at the age of 6 weeks were orthotopically injected with 1×10^6^ Luc‐labeled HCCLM3 cells in the liver. After 14 days, the fluorescence intensity was measured via an in vivo imaging system for random grouping. The cells were divided into three groups: saline, siNC@PD, and siTRIM47@PD. On Days 0, 3, and 6, the mice were intravenously injected with siRNA@PD NPs (1 mg/kg) according to the respective treatment groups. On Day 28, in vivo bioluminescence imaging was conducted, and the bioluminescence signal intensity of the tumors was quantified.

Pharmacokinetic evaluation: male BALB/c nude mice (*n* = 3) were injected with Free siTRIM47^Cy5^ and siTRIM47^Cy5^@PD at a siTRIM47^Cy5^ dose of 1 mg/kg. At 0.5, 1, 2, 4, 8, 12, 24, and 48 h post injection, 50 µL blood was sampled from the orbital vein for siTRIM47^Cy5^ quantification. Briefly, blood was centrifuged at 1500 rpm for 15 min at 4°C. Then, 20 µL serum was dispersed in 100 µL extraction buffer, and vortexed for 30 min. siTRIM47^Cy5^ was then assayed using fluorescent detection (Ex 650 nm, Em 670 nm). The drug concentration versus time plot was drawn.

### 4.13 Bioinformatics Analysis

All bioinformatics analyses were performed using the R language (version 4.1.2). We downloaded mRNA expression data and corresponding clinical information from the TCGA (https://portal.gdc.cancer.gov/) database for HCC patients. mRNA expression data for the Japanese HCC cohort were obtained from the ICGC (https://dcc.icgc.org/). Additionally, mRNA expression data from HCC cohorts (GSE36376, GSE45267, GSE57957, GSE62232, and GSE87630) were retrieved from the GEO (https://www.ncbi.nlm.nih.gov/geo/). When performing correlation data analysis, we excluded cases with missing data. We downloaded the glycolysis/gluconeogenesis gene set “KEGG_GLYCOLYSIS_GLUCONEOGENESIS” from the MSigDB database (https://www.gsea‐msigdb.org/gsea/msigdb). To estimate the levels of glycolysis and gluconeogenesis, ssGSEA was performed using the GSEABase and GSVA packages based on mRNA data from the TCGA‐HCC cohort. Subsequently, we conducted WGCNA focusing on the enrichment levels of glycolysis and gluconeogenesis. WGCNA was performed following our previous studies [47, 52], with a soft‐thresholding power set to 0.9, and modules defined by a minimum of 10 genes and module connectivity. This analysis ultimately identified 10 modules. Furthermore, we performed WGCNA based on HCC tumor development and progression, using similar parameters (soft‐threshold power of 0.9, minimum of 10 genes per module, and module connectivity), which resulted in the identification of 14 modules.

### 4.14 Statistical Analysis

All data are expressed as mean ± standard deviation (SD). Statistical analysis was performed using Prism 9.0 (GraphPad Software, USA) software or SPSS 22.0 (SPSS, USA). Differences between groups were analyzed using Student's *t*‐test. Categorical data were analyzed using the chi‐square test (χ^2^) or Fisher's exact test. Kaplan–Meier analysis was used to assess survival differences among subgroups, and the log‐rank test was used for statistical analysis. Factors influencing prognosis were screened based on univariate Cox regression analysis. *p*‐value < 0.05 was considered statistically significant. ^*^ *p* < 0.05; ^**^ *p* < 0.01; ^***^ *p* < 0.001.

## Conflicts of Interest

The authors declare no conflicts of interest.

## Supporting information




**Supporting File**: advs73880‐sup‐0001‐SuppMat.docx.

## Data Availability

The authors declare that all the data supporting the findings of this study are available within the article and its Supplemental information files. All materials in this article are available upon reasonable request from the corresponding authors.
