# Supplementary material for: TRIM47 Regulates Energy Metabolism via Glycolytic Reprogramming to Drive Hepatocellular Carcinoma Progression and Represents an Efficient Therapeutic Target
Source: Adv Sci (Weinh). 2026 Feb 3;13(17):e16996. doi: 10.1002/advs.202416996 (PMC13042927; doi:10.1002/advs.202416996)
Supplement: Supplementary file 1 — Supporting File: advs73880‐sup‐0001‐SuppMat.docx. [file ADVS-13-e16996-s001.docx]

**Supporting Information**

Supporting Information is available from the Wiley Online Library or from the author.

TRIM47 regulates energy metabolism via glycolytic reprogramming to drive hepatocellular carcinoma progression and represents an efficient therapeutic target

Weijie Sun^1,2,3,#^, Yihang Yuan^4,#^, Qian Qiu^5,#^, Kexuan Tan^1,3,#^, Xutong Li^6,^, Haotian Li^1,3,^, Luyang Kang^1,3,^, Yuting Gu^1,3,^, Ziheng Zhang^7,^, Jiayu He^1,3,^, Jiali Li^1,3,^, Junjie Lin^1,3,^, Zihan Xie^1,3,^, Kexing Han^1,3,^, Jiabin Li^1,3,*^, Yang Zhang^8,*^, Ting Wu^1,3,*^, Yufeng Gao^1,3,*^

*1. Department of Infectious Disease, The First Affiliated Hospital of Anhui Medical University, Hefei, 230000, China*

*2. Department of Medical Oncology, First Affiliated Hospital of Bengbu Medical University, Bengbu, 233000, China*

*3. Anhui Province Key Laboratory of Infectious Diseases, Anhui Medical University, Hefei, 230000, China*

*4. Department of General Surgery, Nanjing Drum Tower Hospital Aﬃliated Hospital of Medical School, Nanjing University, Nanjing, 210008, China*

*5. Department of Pathology, The First Affiliated Hospital of Anhui Medical University, Hefei, 230000, China*

*6. Department of Immunology, School of Basic Medical Sciences, Anhui Medical University, Hefei, 230032, China.*

*7. School of Life Sciences, Jiangsu University, Zhenjiang, 212013, China*

*8. Center for Nanomedicine and Department of Anesthesiology, Perioperative and Pain Medicine, Brigham and Women’s Hospital, Harvard Medical School, Boston, 02115, USA*

*Corresponding Author: Email addresses: lijiabin@ahmu.edu.cn (J. Li); yzhang169@bwh.harvard.edu (Y. Zhang), ORCID: 0000-0002-3476-8937; wutingf88945@163.com (T. Wu); gaoyufeng0917@126.com (Y. Gao).

# These authors made equal contributions to this work.

**Abbreviation and description of different preparations.**

| **Abbreviation** | **Description** |
| --- | --- |
| HCC | Hepatocellular carcinoma |
| TRIM47 | Tripartite motif-containing protein 47 |
| FBP1 | Fructose-1,6-bisphosphatase |
| PTM | Post-translational modification |
| WGCNA | Weighted gene co-expression network analysis |
| siRNA | Small interfering RNA |
| PLA | Poly lactic acid |
| FDA | Food and drug administration |
| DC-Chol | 3β-(N-(N′, N′-dimethylethylenediamine)-carbamoyl) cholesterol |
| PD | PLA and DC-Chol |
| NPs | nanoparticles |
| ATP | Adenosine triphosphate |
| ROS | Reactive oxygen species |
| ssGSEA | Single sample gene set enrichment analysis |
| TCGA | The cancer genome atlas |
| ICGC | International cancer genome consortium |
| DEGs | Differentially expressed genes |
| GEO | Gene expression omnibus |
| PDOs | Patient-derived organoids |
| WB | Western blotting |
| GSVA | Gene set variation analysis |
| IF | Immunofluorescence |
| ECAR | Extracellular acidification rate |
| OCR | Oxygen consumption rate |
| CoIP | Coimmunoprecipitation |
| CHX | Cycloheximide |
| IHC | Immunohistochemical |
| PCNA | Proliferating cell nuclear antigen |
| HE | Hematoxylin-eosin |

**Supplementary Figures**


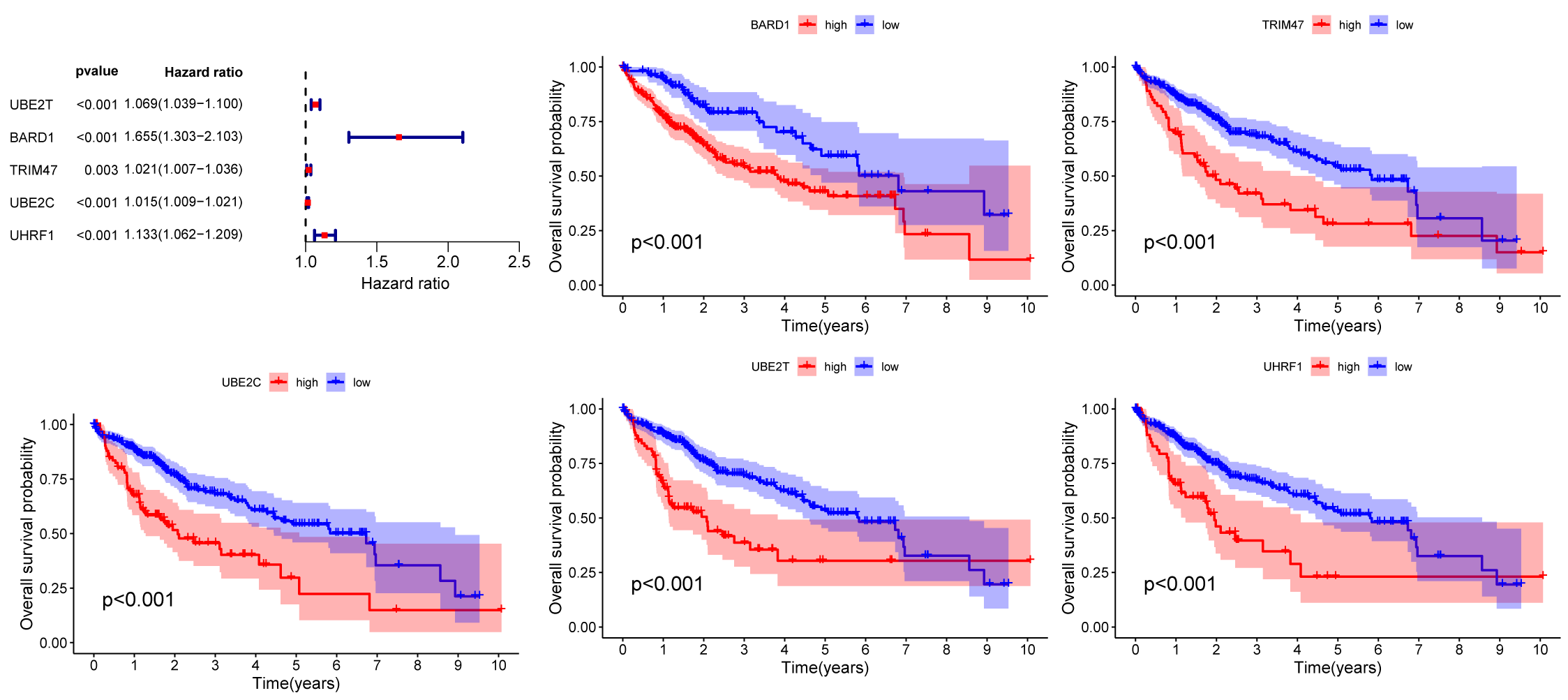


Supplementary Figure 1: Univariate Cox regression analysis and Kaplan-Merier survival curves to evaluate the prognostic value of key genes in the TCGA-HCC cohort


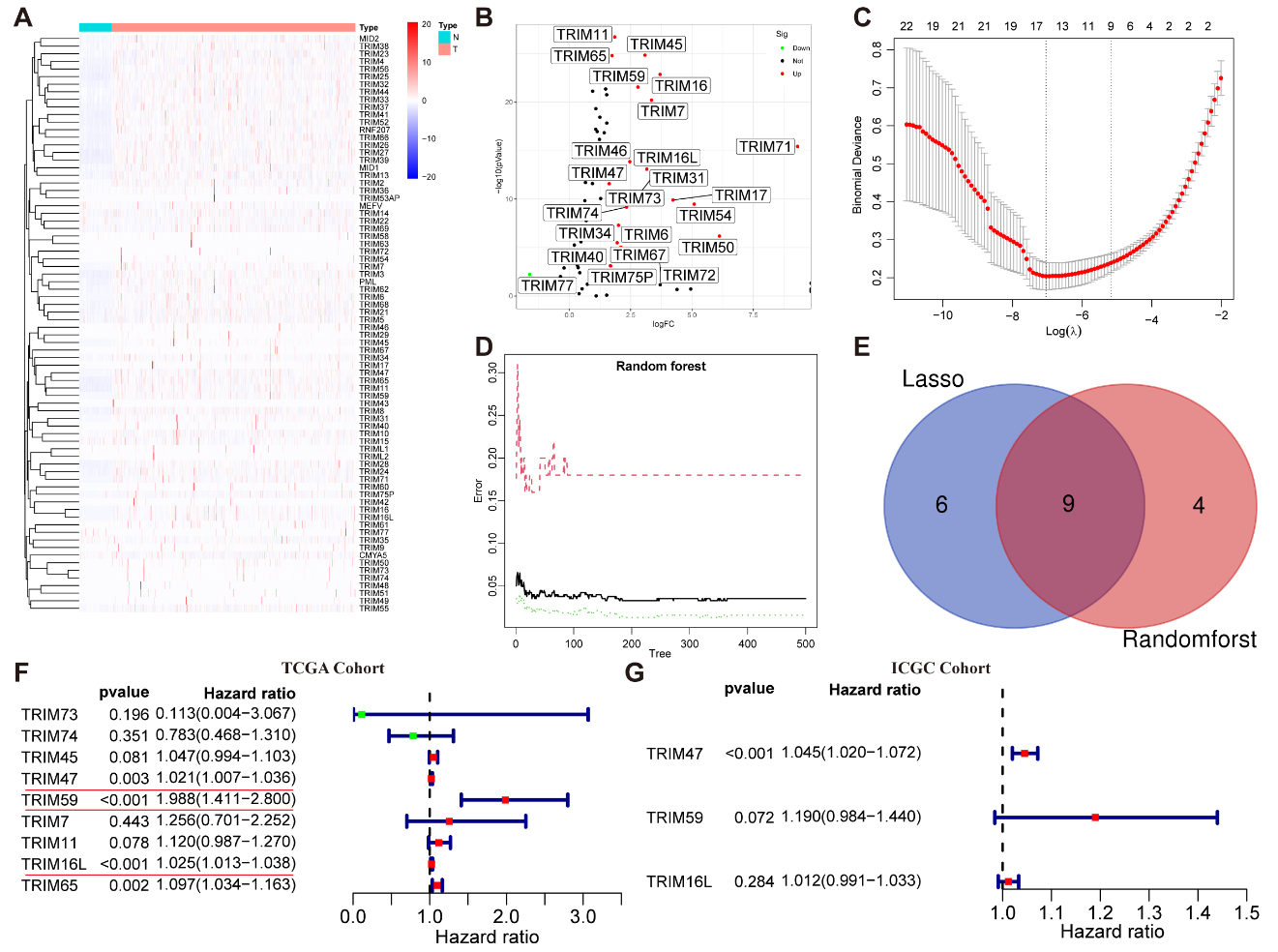


Supplementary Figure 2: Comprehensive analysis of TRIM family genes.

Heat map (A) and volcano plot (B) of differential expression analysis of TRIM family members in the TCGA-HCC cohort. LASSO algorithm (C) and random forest algorithm (D) filter differentially expressed TRIM family members; (E) Venn diagram of the common results of the two machine learning methods. (F) Univariate Cox regression analysis of 9 TRIM family characteristic genes in the TCGA-HCC cohort. (G) Univariate Cox regression analysis of TRIM16, LTRIM47 and TRIM59 in the ICGC cohort.


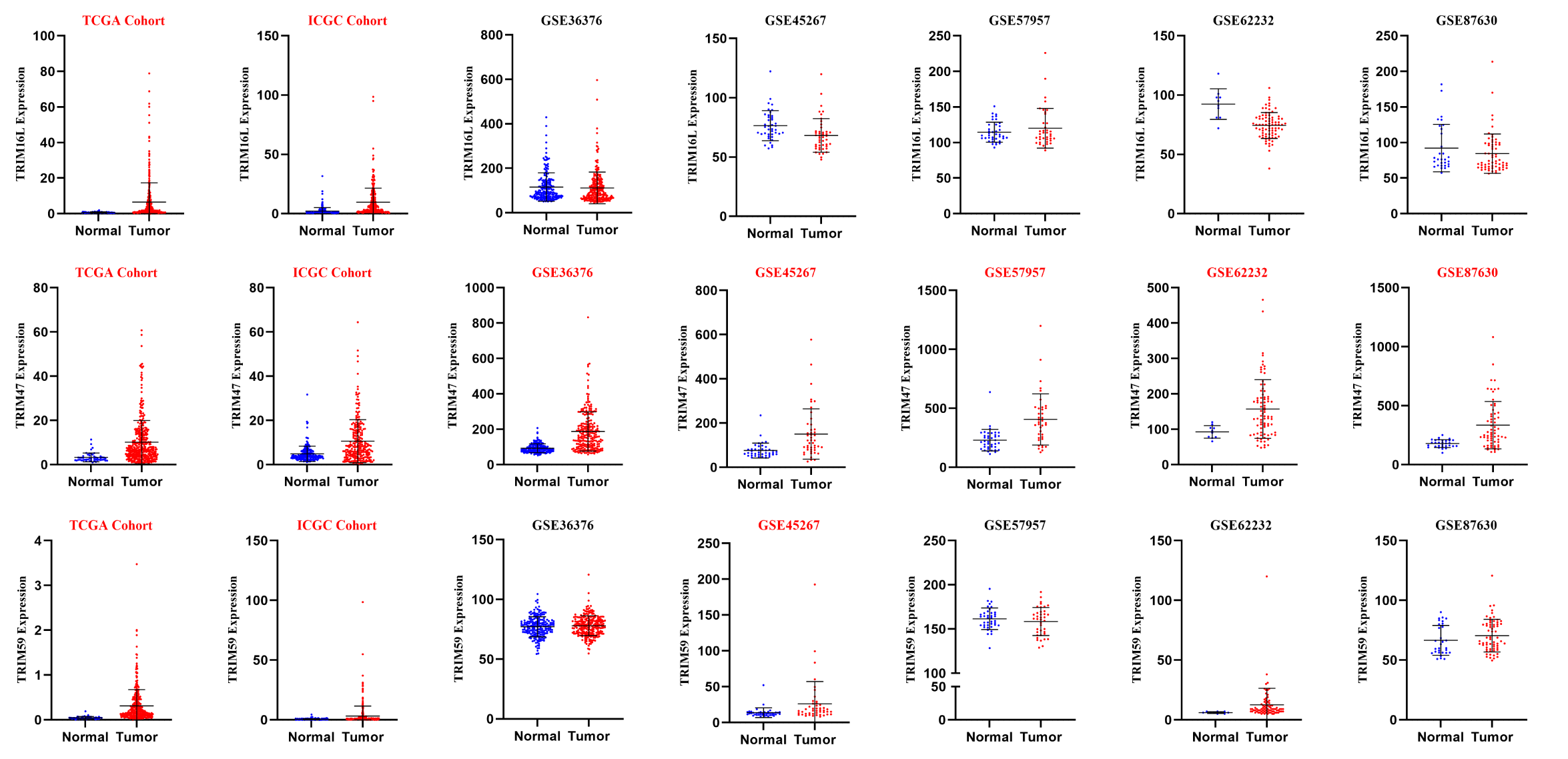


Supplementary Figure 3: Validation of expression patterns of key genes in external cohorts. Differential expression analysis of TRIM16, LTRIM47 and TRIM59 in 6 external liver cancer cohorts. GEO number red indicates statistically significant difference (*p* < 0.05), black indicates no statistical difference (*p* > 0.05).


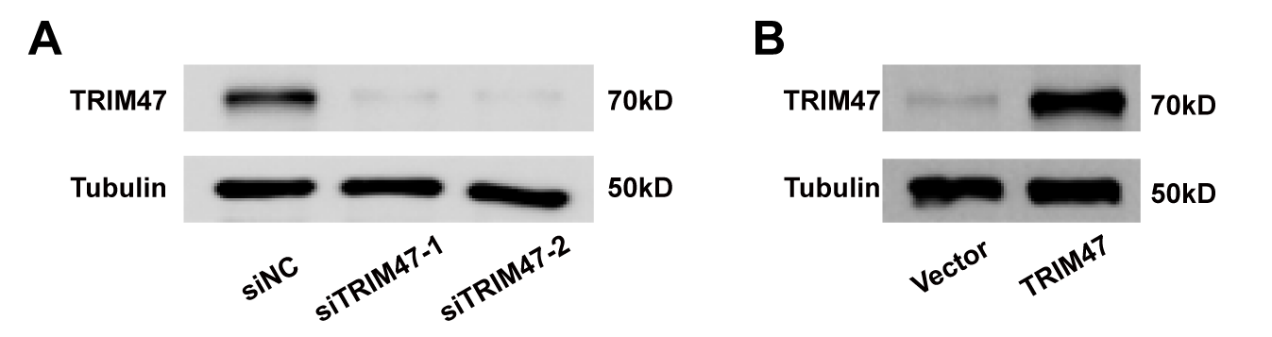


Supplementary Figure 4: Western blotting was used to verify the knockdown and overexpression efficiency of TRIM47 in PDOs.


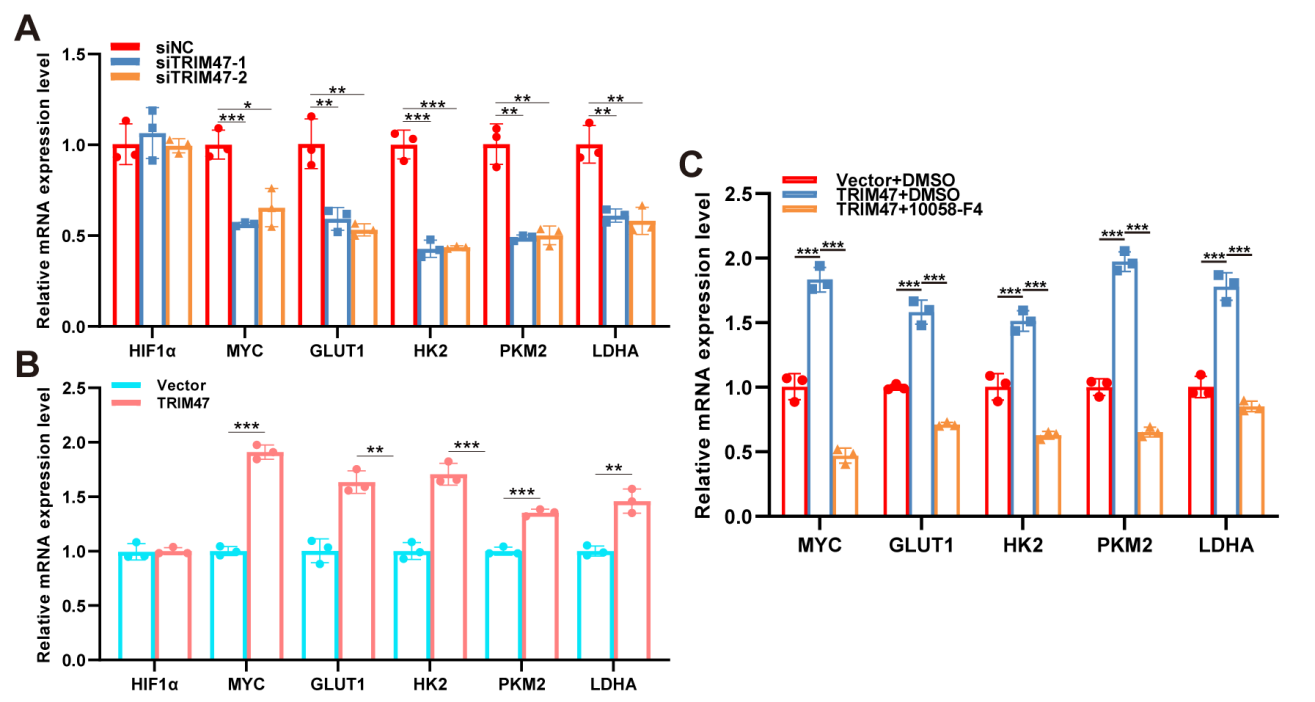


Supplementary Figure 5: The Impact of TRIM47 Expression on Glycolysis-Related Gene Expression. (A) Effect of TRIM47 knockdown on the expression of glycolysis-related genes. (B) Effect of TRIM47 overexpression on the expression of glycolysis-related genes. (C) Effect of TRIM47 overexpression combined with 20 µM 10,058-F4 treatment for 24 hours on glycolysis-related gene expression.. *: p < 0.05; **: p < 0.01; ***: p < 0.001.


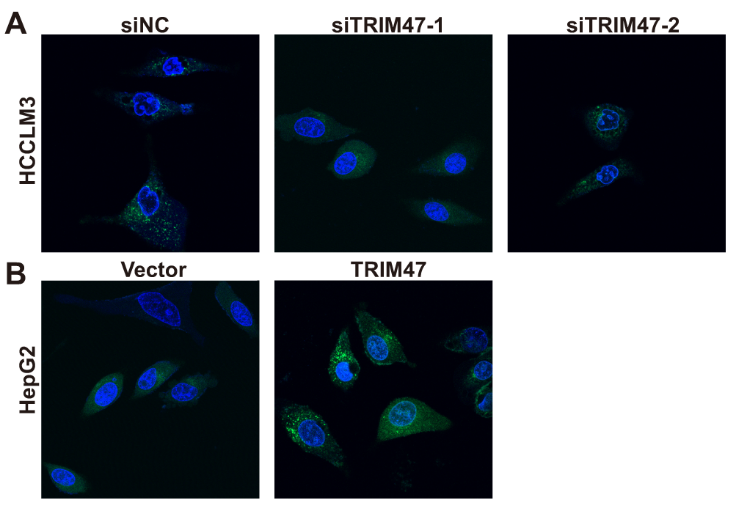


Supplementary Figure 6: Confocal microscopy was used to observe the effect of TRIM47 expression on glucose absorption capacity.


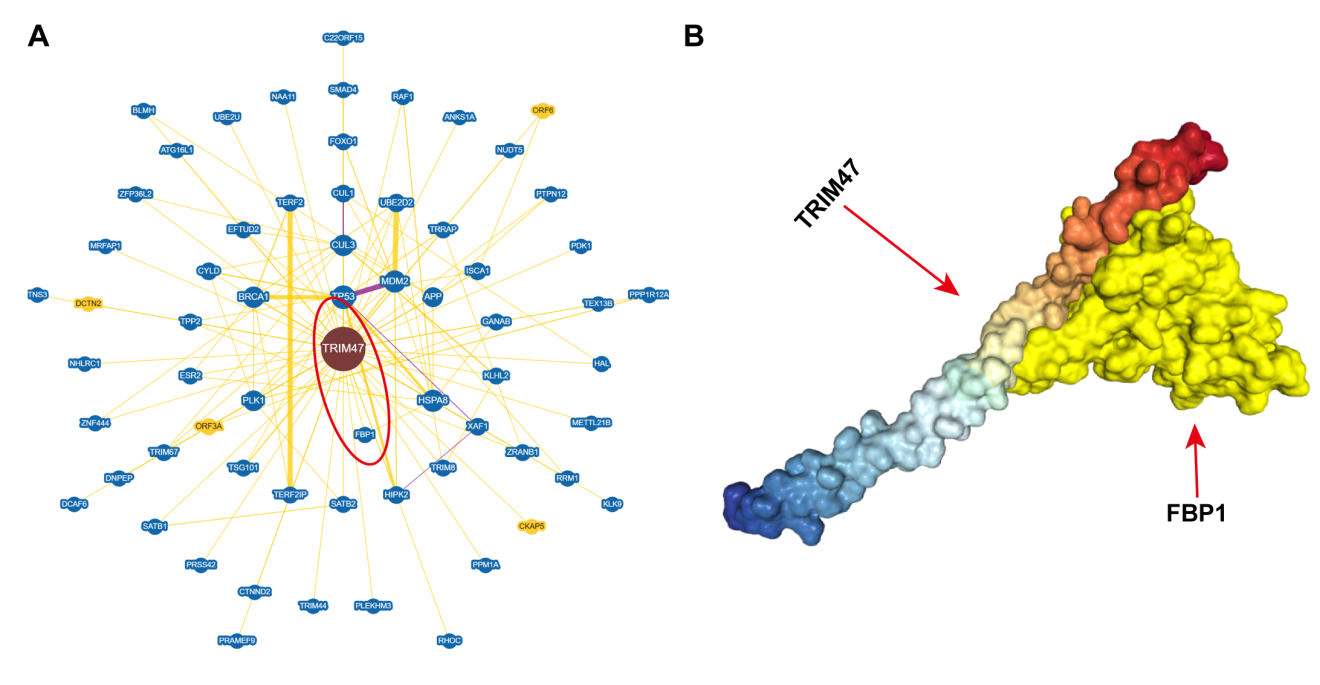


Supplementary Figure 7: Prediction of interaction between TRIM47 and FBP1. (A) Protein network diagram interacting with TRIM47 protein. (B) Docking model between TRIM47 and FBP1.


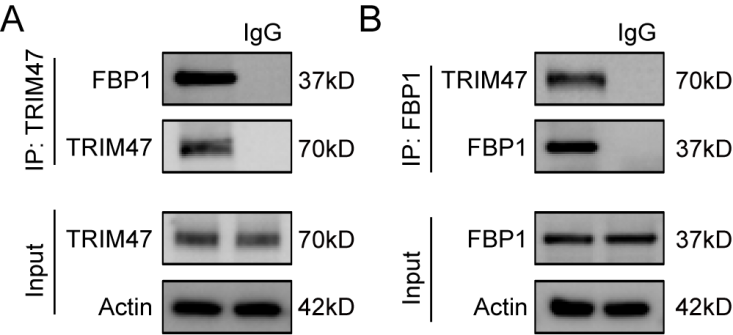


Supplementary Figure 8: CoIP results of endogenous TRIM47 and FBP1 in HEK293T cells.


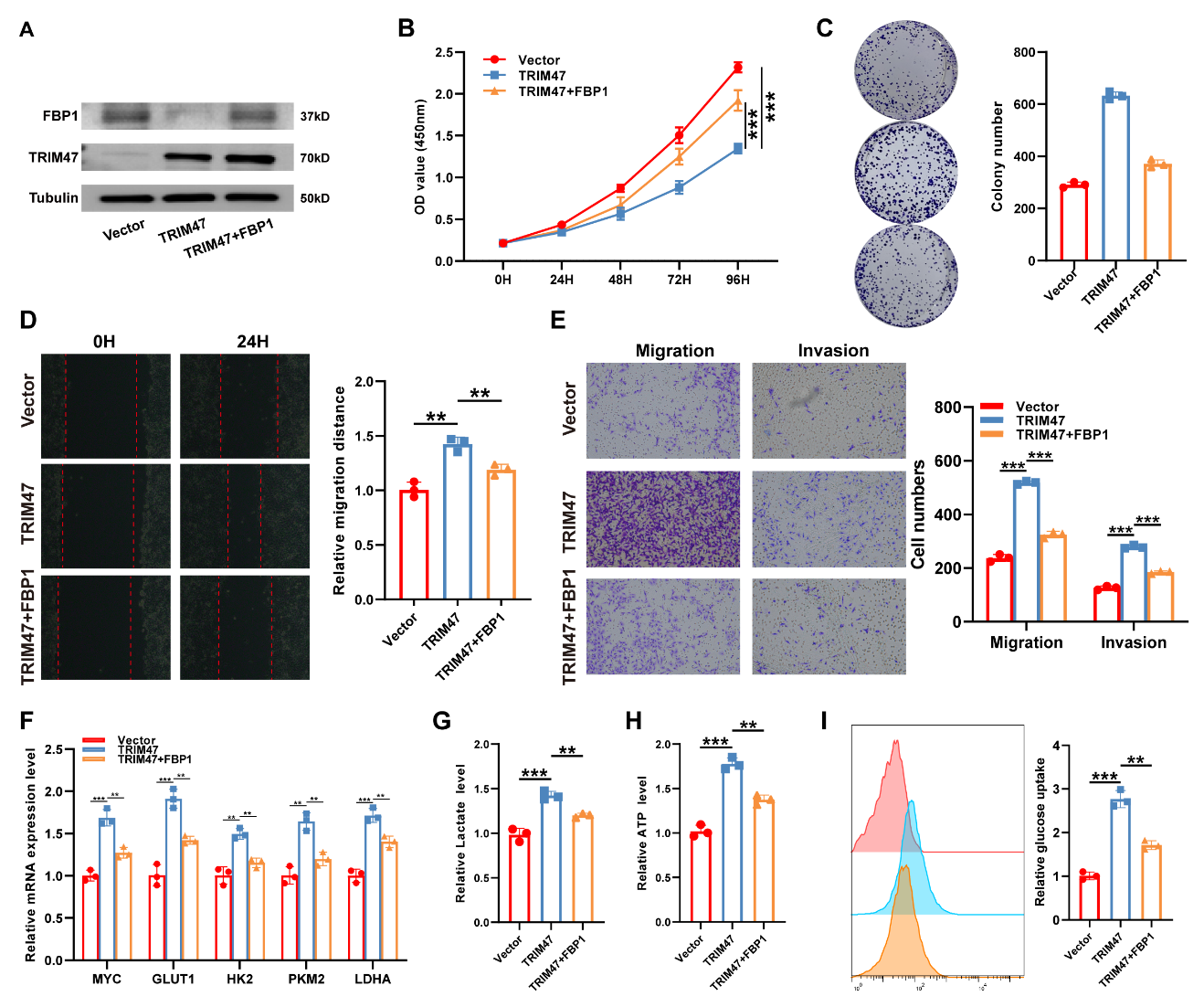


Supplementary Figure 9: FBP1 Mediates the Oncogenic Role of TRIM47 in HCC. (A) Changes in TRIM47 and FBP1 protein levels after transfection of TRIM47 lentivirus and FBP1 plasmid. (B-E) Cell function rescue experiments showed that overexpression of FBP1 could inhibit the enhanced proliferation, migration and invasion of HCC cells induced by siTRIM47. (F-I) Overexpression of FBP1 can inhibit the increase in key glycolysis-related gene expression, lactate production, ATP generation and glucose uptake caused by TRIM47. **：p<0.01; ***：p<0.001.


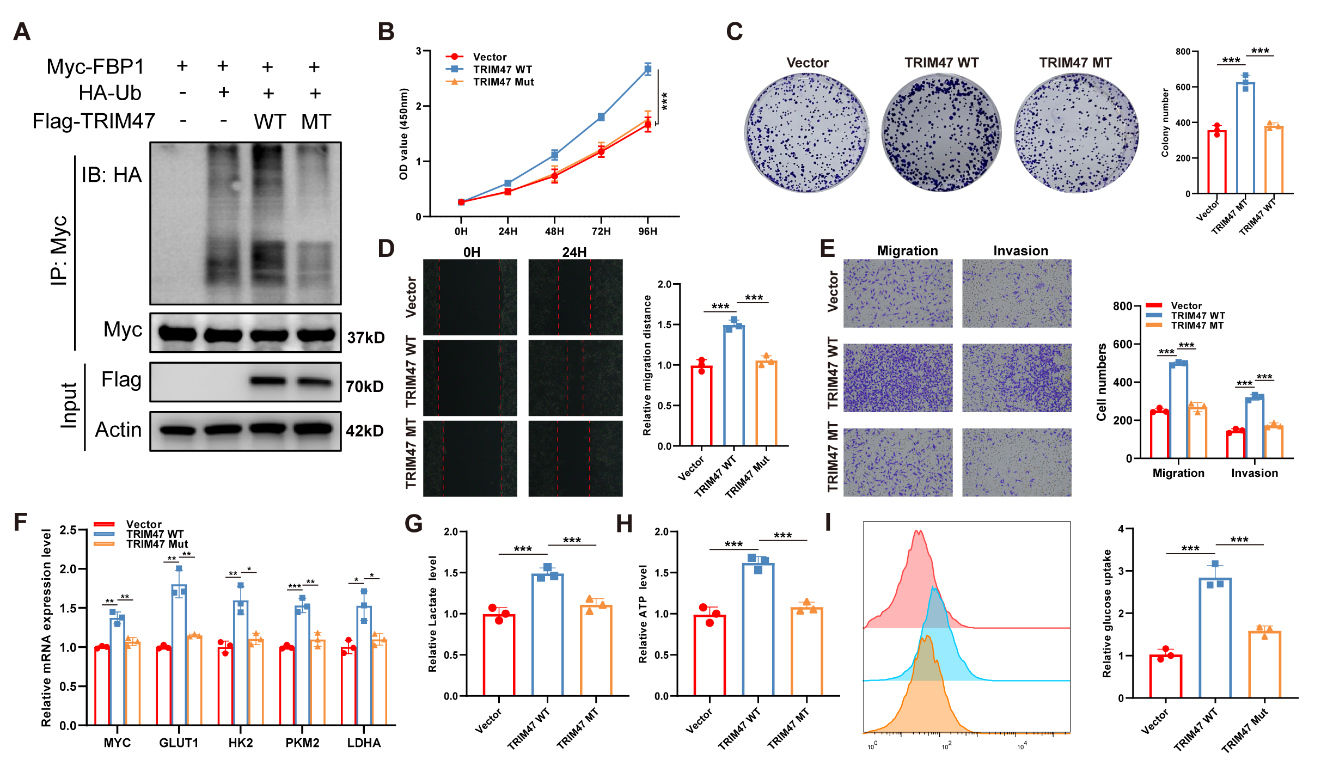


Supplementary Figure 10: TRIM47 regulates FBP1 expression through E3 ubiquitin ligase activity and exerts regulatory glycolysis and oncogenic potential. (A) Changes in the level of FBP1-enriched UB after transfection of TRIM47 WT or TRIM47 MT plasmid. (B-C) Effects of transfection of TRIM47 WT or MT plasmid on the proliferation ability of HCC cells. (D-E) Effects of transfection of TRIM47 WT or MT plasmid on the migration and invasion abilities of HCC cells. (F-I) Changes in the expression of key glycolysis-related genes, lactate production, ATP production, and glucose uptake after transfection with TRIM47 WT or MT plasmids. *：p<0.05; **：p<0.01; ***：p<0.001.


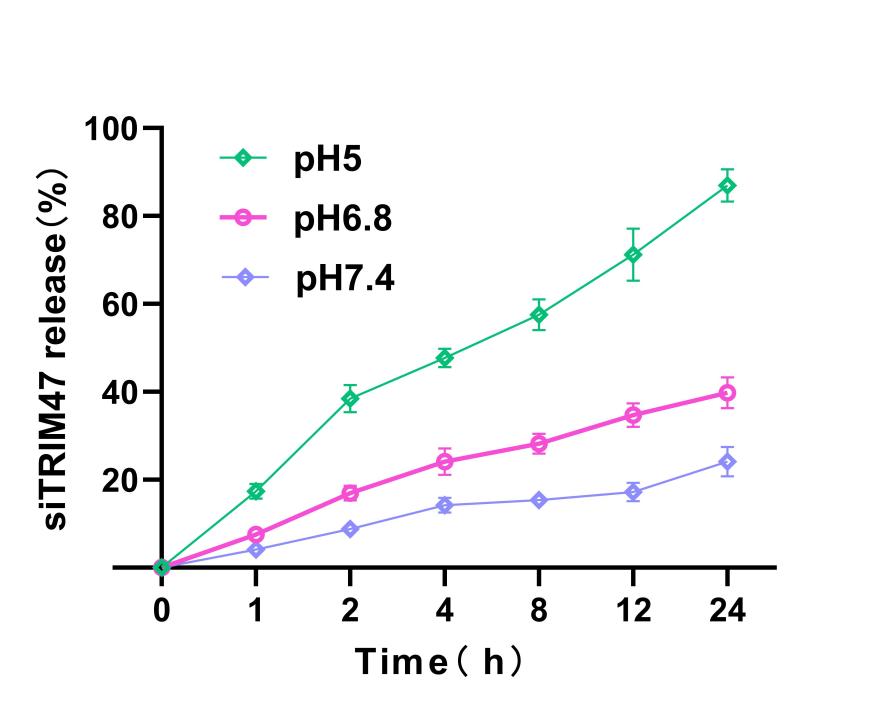


Supplementary Figure 11: In vitro release profile of siTRIM47 from siTRIM47@PD nanoparticles under different pH conditions. Cumulative release of siTRIM47 from PD nanoparticles was measured at pH 5.0, pH 6.8, and pH 7.4 over 24 hours. The nanoparticles exhibited accelerated siRNA release under acidic conditions, indicating pH-responsive release behavior conducive to endosomal escape and intracellular delivery. Data are presented as mean ± SD (n = 3).


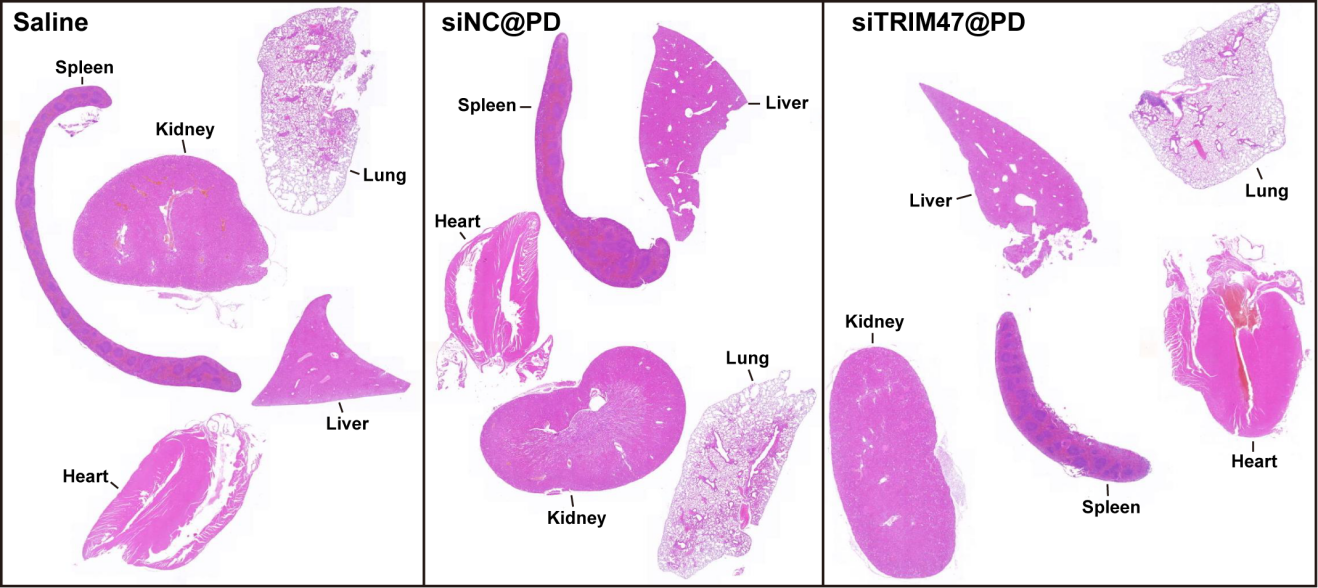


Supplementary Figure 12: HE staining was used to examine the damage of nanoparticle siRNA@PD to important organ tissues.


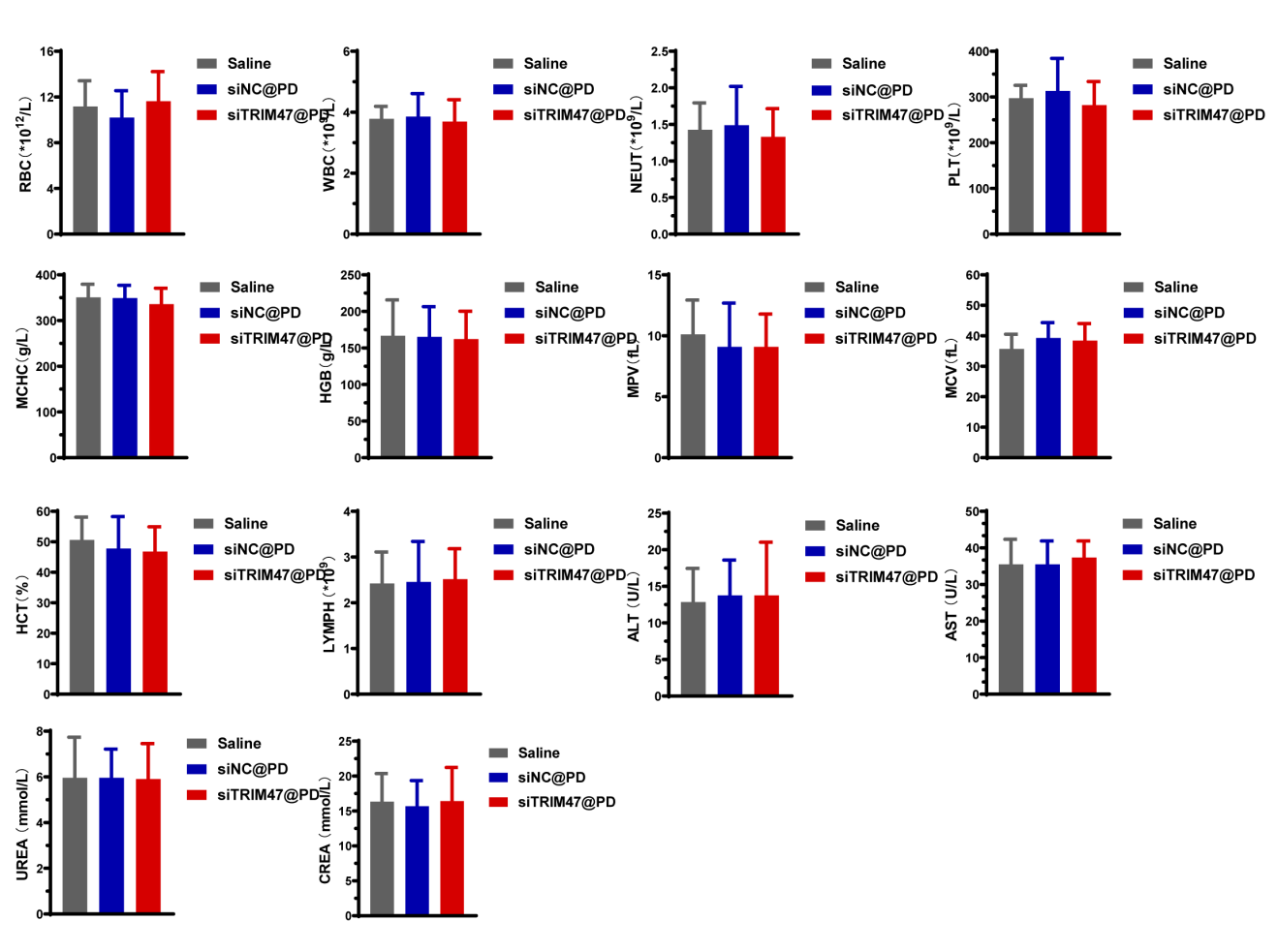


Supplementary Figure 13: Biosafety Assessment of siRNA@PD Nanosystem. Hematological and biochemical analyses showing no significant toxicity after treatment with saline, siNC@PD, or siTRIM47@PD in mice.

Supplementary Figure 14: Pharmacokinetic analysis of nanoparticle siTRIM47@PD in mice.

*Supplementary Table 1*: Distribution of clinical pathological features of TRIM47 in HCC.

| Clinicopathological characteristics | IHC scoring of TRIM47 | | p Value |
| --- | --- | --- | --- |
|  | High (N=36) | Low (N=36) |  |
| **Gender** |  |  | 0.527 |
| Male | 29 | 31 |  |
| Female | 7 | 5 |  |
| **Age** | 62（55，65） | 58（48.75，63） | 0.143 |
| **Tumor TNM staging** |  |  | **0.013** |
| I+II | 23 | 32 |  |
| III+IV | 13 | 4 |  |
| **Tumor grade** |  |  | 0.293 |
| G1 | 4 | 9 |  |
| G2 | 20 | 18 |  |
| G3 | 12 | 9 |  |

Note: The 8th edition of the AJCC standard was used for TNM staging of tumors.

Supplementary Table 2: siRNA and shRNA sequences involved in the study

| Gene name | sense | antisense |
| --- | --- | --- |
| siTRIM47-1 | CCUGAAGCUGACUCAGUCA(dT)(dT) | UGACUGAGUCAGCUUCAGG(dT)(dT) |
| siTRIM47-2 | GCCUUCAGCUCCGCAAGAA(dT)(dT) | UUCUUGCGGAGCUGAAGGC(dT)(dT) |
| siFBP1 | CCUUGAUGGAUCUUCCAACAU(dT)(dT) | AUGUUGGAAGAUCCAUCAAGG(dT)(dT) |
| shTRIM47 | CCGGTACTGGGAGGTGGAGATTATCCTCGAGGATAATCTCCACCTCCCAGTATTTTTT | |

*Supplementary Table 3*: qRT-PCR primer sequences

| Gene name | Forward Sequence | Reverse Sequence |
| --- | --- | --- |
| GAPDH | GTCTCCTCTGACTTCAACAGCG | ACCACCCTGTTGCTGTAGCCAA |
| TRIM47 | CTGACTCAGTCAGCTTCCTGCA | TCTCTCACTGCACGGACAGCTT |
| FBP1 | GCAGTCAAAGCCATCTCTTCGG | TAACCAGGTCGTTGGAGAGGAC |
| HIF1-α | TATGAGCCAGAAGAACTTTTAGGC | CACCTCTTTTGGCAAGCATCCTG |
| c-Myc | CCTGGTGCTCCATGAGGAGAC | CAGACTCTGACCTTTTGCCAGG |
| GLUT1 | TTGCAGGCTTCTCCAACTGGAC | CAGAACCAGGAGCACAGTGAAG |
| HK2 | GAGTTTGACCTGGATGTGGTTGC | CCTCCATGTAGCAGGCATTGCT |
| PKM2 | ATGGCTGACACATTCCTGGAGC | CCTTCAACGTCTCCACTGATCG |
| LDHA | GGATCTCCAACATGGCAGCCTT | AGACGGCTTTCTCCCTCTTGCT |

*Supplementary Table 4*: Antibodies used in Western blotting and Immunofluorescence

| Name | Sourse |
| --- | --- |
| β-tublin | https://www.ptgcn.com/ |
| Actin | https://www.ptgcn.com/ |
| TRIM47 | https://www.ptgcn.com/ |
| HA | https://www.ptgcn.com/ |
| Flag | https://www.ptgcn.com/ |
| Myc | https://www.ptgcn.com/ |
| FBP1 | https://www.ptgcn.com/ |
| G6PC | https://www.ptgcn.com/ |
| PCK2 | https://www.ptgcn.com/ |
| PC | https://www.ptgcn.com/ |
| p-mTOR | https://www.cellsignal.cn/ |
| mTOR | https://www.cellsignal.cn/ |
| p-MAPK | https://www.cellsignal.cn/ |
| MAPK | https://www.cellsignal.cn/ |
| p-AKT | https://www.cellsignal.cn/ |
| AKT | https://www.cellsignal.cn/ |
| Anti-rabbit secondary antibody | https://www.ptgcn.com/ |
| Anti-mouse secondary antibody | https://www.ptgcn.com/ |
| Fluorescent secondary antibody | https://www.ptgcn.com/ |
